# Microbiome succession with increasing age in three oral sites

**DOI:** 10.18632/aging.103108

**Published:** 2020-05-07

**Authors:** Shili Liu, Yihua Wang, Le Zhao, Xiaoyuan Sun, Qiang Feng

**Affiliations:** 1 Department of Human Microbiome, School and Hospital of Stomatology, Cheeloo College of Medicine, Shandong University and Shandong Provincial Key Laboratory of Oral Tissue Regeneration and Shandong Engineering Laboratory for Dental Materials and Oral Tissue Regeneration, Jinan 250012, Shandong, China; 2 School of Basic Medical Science, Cheeloo College of Medicine, Shandong University, Jinan 250012, Shandong, China; 3 State Key Laboratory of Microbial Technology, Shandong University, Qingdao 266237, Shandong, China; 4 Shandong University Hospital, Jinan, Shandong 250100, China

**Keywords:** oral microbiota, age-differentiated bacteria, oral sites, core microbiome, biomarker

## Abstract

The incidence of oral diseases is remarkably increased with age, and it may be related to oral microbiota. In this study, we systematically investigated the microbiota of gingival crevicular fluid (GCF), tongue back (TB) and saliva (SAL) from various age groups in healthy populations. The microbial diversity results indicated that the α-diversity of bacteria had a tendency to decrease in aging mouth, whereas the β-diversity showed an opposite increasing trend in all three sites. Next, the microbial structure exploration revealed a divergence in bacterial profile in three sites in response to aging, but the intersite differential bacteria demonstrated a uniform bell-shaped variation trend with age. Meanwhile, several age-differentiated genera were shared by GCF, SAL and TB sites, and the bacterial correlation analysis demonstrated a clear shift in the pattern of bacterial correlations with age. Moreover, both the intra- and intersite “core microbiome” showed significantly decreased bacterial diversities with age. Finally, the trending differential bacteria species were used as a biomarker to distinguish the different age groups, and the prediction accuracies in GCF were 0.998, 0.809, 0.668, 0.675 and 0.956. Our results revealed the characteristics of intra- and intersite bacterial succession with age, providing novel insights into senile oral diseases.

## INTRODUCTION

With the continuous development of society, the proportion of the elderly in the population is continuously increasing year by year. At the beginning of this century, there were approximately 600 million people over the age of 60 worldwide, and this number is expected to double by 2025 [1]. In the elderly, the incidence of oral diseases such as dental caries, periodontal disease, and soft tissue disorders is generally higher than in younger people [2, 3]. The reasons may be due to oral tissue degeneration and oral microbial infection [4]. For example, the salivary glands of the elderly will shrink, reduce the secretion of saliva, and change the composition and growth status of the dominant bacteria [5]. However, the variation in oral microbiota in the elderly has not been extensively studied.

The microbiota that resides in the oral cavity constitutes an ecological environment [6], and the incidence of oral diseases is closely associated with alteration of the oral microbiome. The occurrence of oral diseases varies significantly at different ages, especially the high incidence of multiple oral diseases in the elderly population, suggesting that the oral flora may change with age and be related to disease occurrence. Some studies have detected changes in certain bacteria with age. Percival et al. studied the effects of aging on the composition of oral microbiota in individuals with healthy periodontium [7], and their results showed that Actinomycetes spp., especially *Actinomyces naeslundii* and *Actinomyces oris*, were significantly higher in the supragingival biofilm of subjects over 60 years of age. Preza et al. investigated bacterial profiles in the oral cavity of elderly people who have no caries and periodontitis [8], and they found that older people might have higher bacterial diversity than young and middle-aged adults. Rodenburg et al. studied patients with periodontitis in four age groups and found that the prevalence of periodontitis in subjects colonized by *Aggregatibacter actinomycetemcomitans* decreased with age, whereas those colonized by *Porphyromonas gingivalis* increased with age [9]. Slots et al. reported a higher prevalence of enteric bacilli and Pseudomonas species in older subjects than in younger individuals [10]. After the new 16S ribosomal RNA-based taxonomic survey method was applied, studies have shown that the oral microbial structure changes constantly during certain life stages, such as infant growth [11, 12]. Discovering the pattern of changes in flora with age may enable microbes to have an application value in disease diagnosis and prognosis [13, 14]. However, age-related changes in oral microbiome in a given oral site (such as gingival crevicular fluid and tongue dorsum) have not been adequately addressed, and more extensive work needs to be conducted.

Investigating the characteristics of microbial communities in different sites of the oral cavity with age and exploring how oral bacteria colonize, thrive and decline with age will help with understanding the characteristics of the interaction between microbes and hosts and then answer basic questions about microbial dynamics [15–17]. In the present study, we systematically investigated the microbiota profiles of gingival crevicular fluid (GCF), tongue back (TB) and saliva (SAL) from young (11-15 years old) to old (>50 years old) individuals in healthy populations. Our results reveal intra- and intersite bacterial variation in multiple oral sites over age and provide novel insights into the possible causes of bacterial changes.

## RESULTS

### Characteristics of recruited participants

We collected samples from the gingival crevicular fluid (GCF), saliva (SAL) and tongue back (TB) sites of healthy people of different ages. The subjects were divided into five age groups: Group A (age 11-15), Group B (age 18-20), Group C (age 28-32), Group D (age 38-45) and Group E (age 50–65) (Supplementary Table 1), representing adolescents, youth, the middle-aged and the elderly. All samples were analyzed by 16S rRNA sequencing; detailed information is introduced in the Material and Methods section. After quality filtering, more than 15.05 million clean reads were harvested corresponding to a mean of 84,090 effective tags and 372 OTUs per sample (Supplementary Tables 2 and 3). The rarefaction curve indicated that the OTUs of all samples tended to saturate as the sequence number increased (Supplementary Figure 1), indicating that the OTUs in the data cover most of the reads in the samples.

### Lower within-sample and higher between-sample microbial diversities in aging mouth

**Figure 1:**
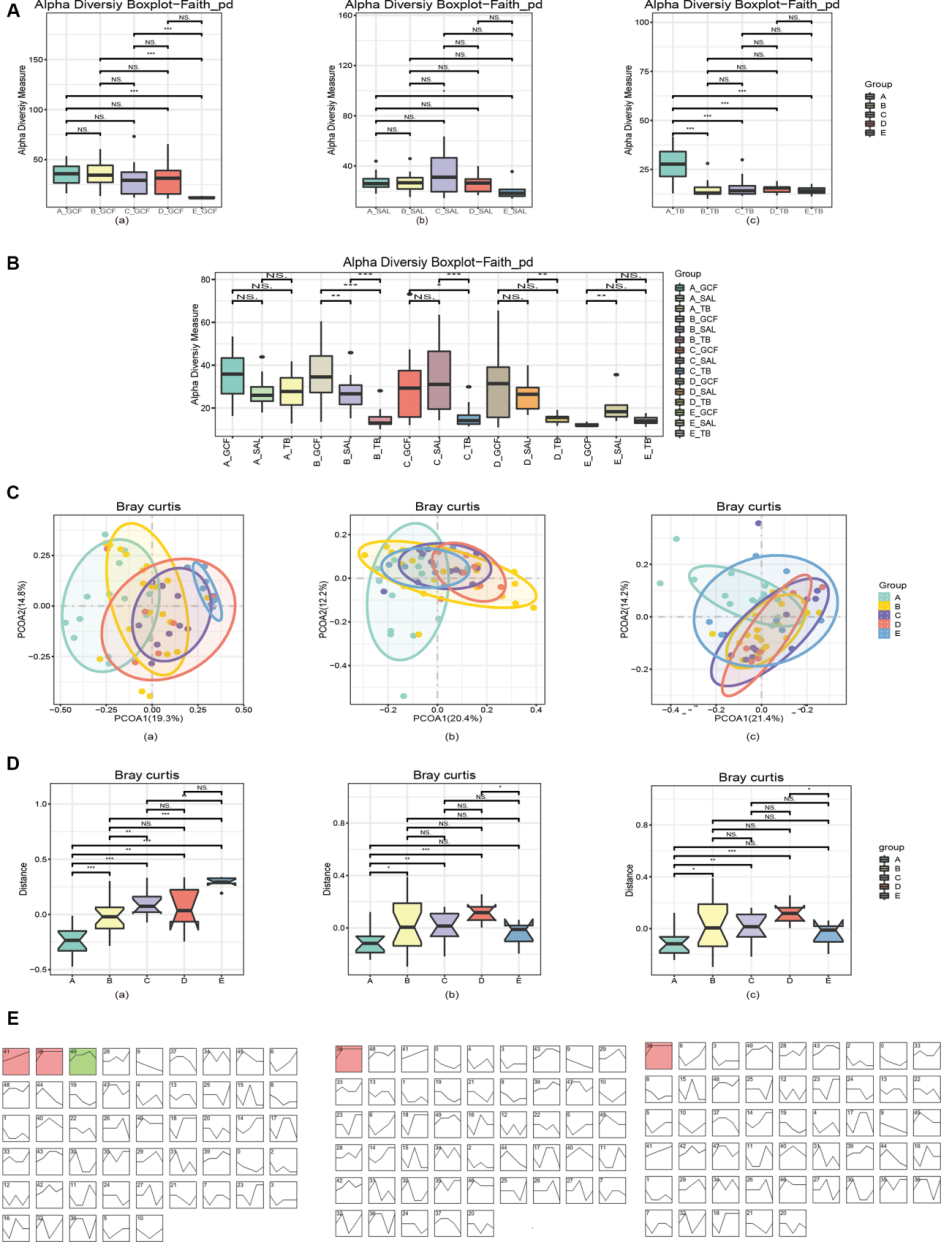
**Diversities of the oral microbiota.** (**A**) The α-diversity indexes of bacteria changed with age in various oral sites. (**B**) The α-diversity indexes of bacteria diverged in different oral sites within each age group. (**C**) The β-diversity indexes of microbiota changed with age, and the pattern varied in various oral sites. (**D**) The first principal component analysis revealed microbial changes with age. (**E**) STEM analysis of the trends of bacterial change over time. In (**A**) and (**C**–**E**), (**a**) GCF; (**b**) SAL; (**c**) TB.

To compare the differences in oral microbiota among different age groups, the α-diversity of the oral microbiota relative to age and oral site was first explored. Microbial OTUs within each oral site were analyzed by ANOVA, and the results showed that the OTUs within GCF or TB had marked reductions with age (Supplementary Figure 2, p=0.0029 and 0.0001, respectively). Meanwhile, the bacterial OTUs in GCF decreased gradually with age, whereas there was no difference between groups B-E for TB; however, the OTU was significantly larger in group A than in the older groups. The Faith_PD diagrams also demonstrated the distinction of microbial within-sample diversity between different age groups within each site (Figure 1A). The variation trends of Faith_PD in GCF, SAL and TB were similar to that of OTUs. Additionally, an intersite comparison was performed on the bacterial α-diversity within each age group, and the cross-site pattern of Faith_PD revealed differentiation in α-diversity between various oral sites. The α-diversity differed between at least two sites except group A (Figure 1B), further demonstrating the distinction of bacteria in different oral sites and age-related variation.

Subsequently, the β-diversity (between-sample) of the oral microbiota in response to aging was investigated. The principal coordinate analysis (PCoA) by the Bray_Curtis distance illustrated a remarkable shift in microbial distribution between age groups (Figure 1C), and the p-values obtained by ADONIS (permutational MANOVA) analysis of the bacterial variations in GCF, SAL and TB were <0.001, 0.003 and <0.001, respectively. The comparison of the first principal components by Bray_Curtis showed that microbial β-diversity significantly increased with age (Figure 1D), except for that of SAL and TB, which declined in the >50 years group. Furthermore, the principal coordinate analysis (PCoA) by the Bray_Curtis distance illustrated the cross-site variation in microbial distribution, and the p-values obtained by ADONIS (permutational MANOVA) analysis were <0.001 for Group A-D and 0.036 for Group E (Supplementary Figure 3). The first principal components analysis by Bray_Curtis also showed the β-diversity differed at least between two sites (Supplementary Figure 4).

To verify the changes in between-sample diversities, the software STEM (Short Time-series Expression Miner) was used to cluster bacteria into modules and calculate their p-values. The results showed that 3, 1 and 1 microbial clusters had a p-value of <0.001 (non-random) in GCF, SAL and TB, respectively (Figure 1E). Analyzing the tendency of variation in each module, the results revealed a rising trend with various patterns. Meanwhile, the TB and SAL modules increased sharply at first but then slowly while a module at the GCF site decreased in the last age group (Figure 1E and Supplementary Figure 5, Supplementary Table 4). Checking the tendency of variation and bacterial composition of each module, *Eubacterium sulci, Stomatobaculum longum, Alloprevotella rava, Porphyromonas gingivalis, Abiotrophia defectiva, Porphyromonas endodontalis, Parvimonas micra,* and *Dialister pneumosintes* increased their abundance with age in GCF*; Dialister pneumosintes, Tannerella forsythia, Treponema medium,* and *Slackia exigua* increased their abundance with age in saliva; and only *Alloprevotella rava* was found with enhanced abundance in TB. In summary, the above results indicate that α-diversity has a tendency to decrease with age, and a cross-site distinction existed. The β-diversity has an increasing trend in three sites, and some bacteria contributed to the increase in β-diversity.

### The alteration of oral microbiota with the increase in age

**Figure 2:**
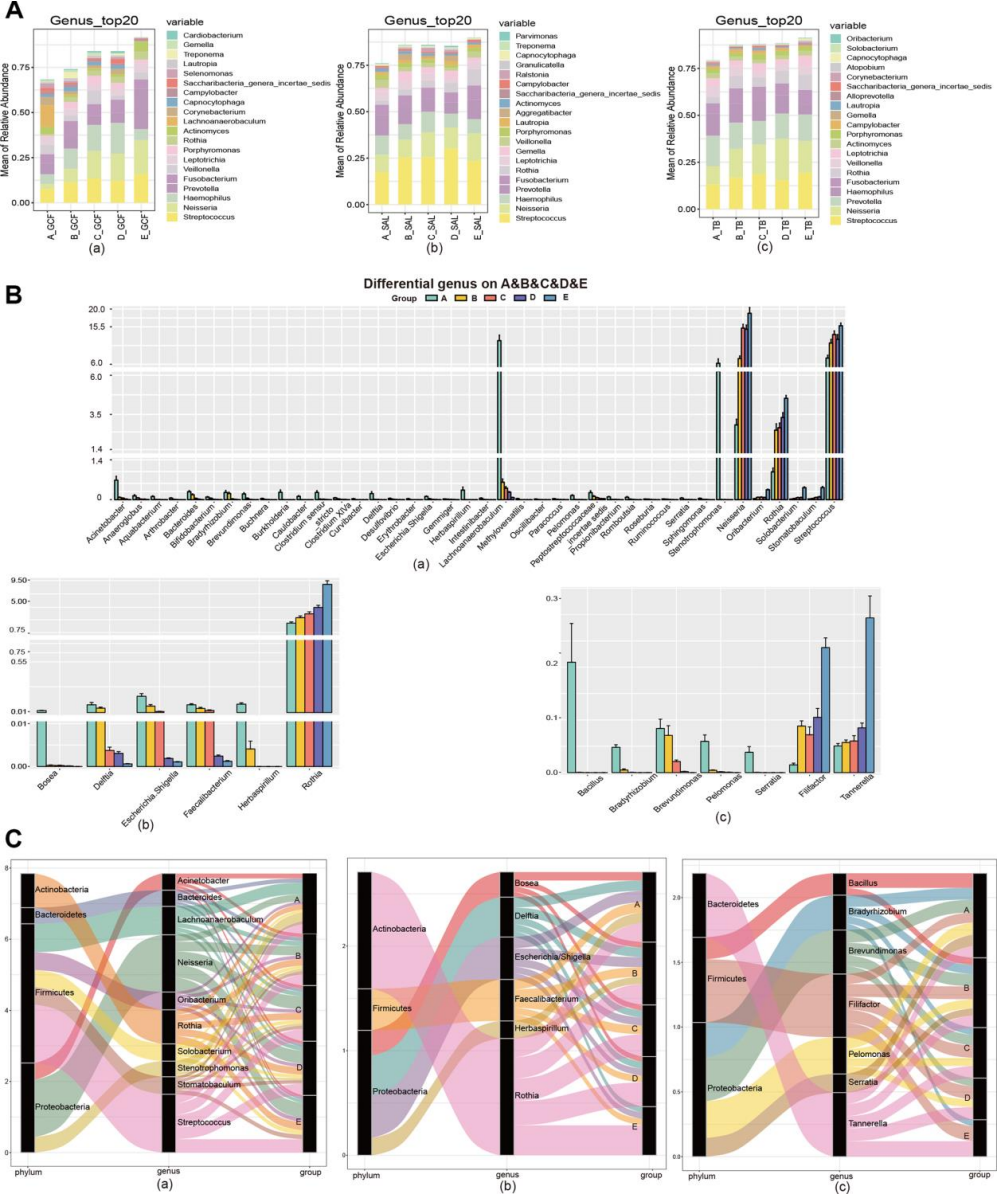
**The community variation of oral microbiota with age.** (**A**) The top 20 genera of the microbiota from the GCF, SAL and TB sites. (**B**) The genus gradually increased or decreased their contents with age. (**C**) The phyla associated with the different genera with high abundance. (**a**) GCF; (**b**) SAL; (**c**) TB.

**Figure 3:**
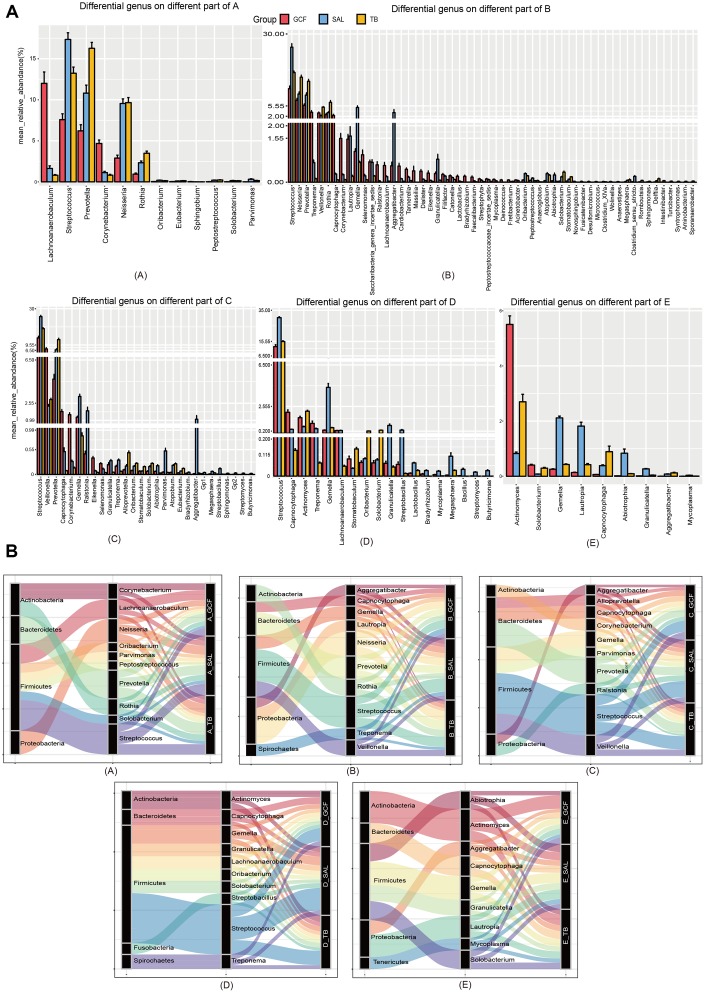
**The community variation of oral microbiota with age.** (**A**) The genera differentially distributed in GCF, SAL and TB. (**B**) The phyla associated with the abundant genera differently distributed in the three oral sites. (**A**–**E**), age groups.

To clarify the effects of age on oral microbiota, we then compared the structure of oral microbiome at the genus and species levels. At the genus level, Streptococcus, Neisseria, Haemophilus, Prevotella and Fusobacterium were dominant in all three sites (Figure 2A). Comparing the abundance of bacterial genera between the age groups, 88, 44 and 47 genera were found to have marked variation with age in the GCF, SAL and TB sites, respectively (Supplementary Figure 6). Meanwhile, 39, 6 and 7 genera enhanced or reduced their contents continuously with age in the GCF, SAL and TB sites, respectively (Figure 2B and Supplementary Figure 7, Supplementary Table 5); i.e., their trends were not changed in the age groups. Among all the genera shifting up or down gradually in all three sites, Delftia, Escherichia/Shigella, and Herbaspirillum were reduced in both the GCF and SAL sites while the Rothia content increased in the two sites. Similarly, Bradyrhizobium, Brevundimonas, Pelomonas and Serratia decreased their abundance with age in both the GCF and TB sites (Figure 2B and Supplementary Table 5). There was no overlap between the SAL and TB sites. At the species level, *Haemophilus parainfluenza, Veillonella dispar, Porphyromonas catoniae* and *Prevotella jejuni* were dominant, with *Haemophilus parainfluenza* having the highest abundance in all three sites (Supplementary Figure 8). Among the species of bacteria included in all age groups, 75, 24 and 49 species were found to have significant age-related variation in the GCF, SAL and TB sites, respectively (Supplementary Figure 9). Meanwhile, 14, 2 and 4 species varied continuously with upward or downward trends in the GCF, SAL and TB sites, respectively (Supplementary Figure 10 and Supplementary Table 5). In GCF, *Acinetobacter* spp., *Actinomyces* spp*.*, *Anaeroglobus geminatus, Bifidobacterium longum* subsp*. infantis, Propionibacterium acnes, Pseudomonas beteli, Sphingomonas xenophagum, Treponema maltophilum, Oribacterium sinus, Prevotella nanceiensis, Solobacterium moorei,* and *Stomatobaculum longum* changed their contents with age continuously*;* in SAL, *Faecalibacterium prausnitzii* and *Fusobacterium mortiferum* had a trend of variation; and in TB, *Eubacterium sulci, Feacalibacterium prausnitzii, Porphyromonas gingivalis* and *Tannerella forsythia* changed their contents gradually (Supplementary Table 5). Among these species with trends, only *Feacalibacterium prausnitzii* decreased in abundance with age in more than one site (SAL and TB) (Supplementary Table 5). The phyla to which the different genera belong were displayed by the Sankey diagram, and the results demonstrated that the different genera harboring GCF and SAL with prevalence all belong to the phyla Firmicutes and Proteobacteria (Figure 2C); meanwhile, other genera in TB belonging to Bacteroidetes were also found. The above results suggest that the microbial composition changes with age, and the patterns of variation in different oral sites were divergent. Afterward, we performed an intersite comparison of the microbial community within each age group at the genus and species levels. At the genus level, 12, 56, 28, 18 and 9 genera were differentially distributed across the sites for the age groups A to E, respectively (Figure 3A and Supplementary Figure 11, Supplementary Table 6); the number of differential genera demonstrated a trend of a bell-shaped curve (increasing first and then decreasing) with age (Figure 3A and Supplementary Figure 11). The cross-site differential genera included Prevotella, Rothia, Solobacterium, etc., but only Solobacterium was shared by all age groups (Supplementary Figure 12, Supplementary Table 6). At the species level, 10, 72, 43, 17 and 25 species showed significantly varied intersite distribution with age, respectively (Supplementary Figures 13, 14, Supplementary Table 6). The number of cross-site differential species also formed a bell-shaped curve in aging mouth. The intersite differential species included *Prevotella* spp., *Actinomyces* spp., and *Solobacterium* spp., etc., but only *Actinomyces odontolyticus* and *Solobacterium moorei* were shared by all age groups (Supplementary Figure 12 and Supplementary Table 6). The phyla to which the intersite differential genera belong were displayed by the Sankey diagram, and the results demonstrated that the different genera in Group A all belonged to the phyla Actinobacteria, Firmicutes and Proteobacteria (Figure 3B), whereas more differential genera belonged to the phyla Bacteroidetes and Teneriquetsas as the host age increased. The results above indicate the separation in the patterns of bacterial composition in various sites of aging mouth, and the number of intersite differential bacteria followed bell-shaped trend in response to aging.

### Variation of the “core microbiome” in GCF, SAL and TB of aging mouth

**Figure 4:**
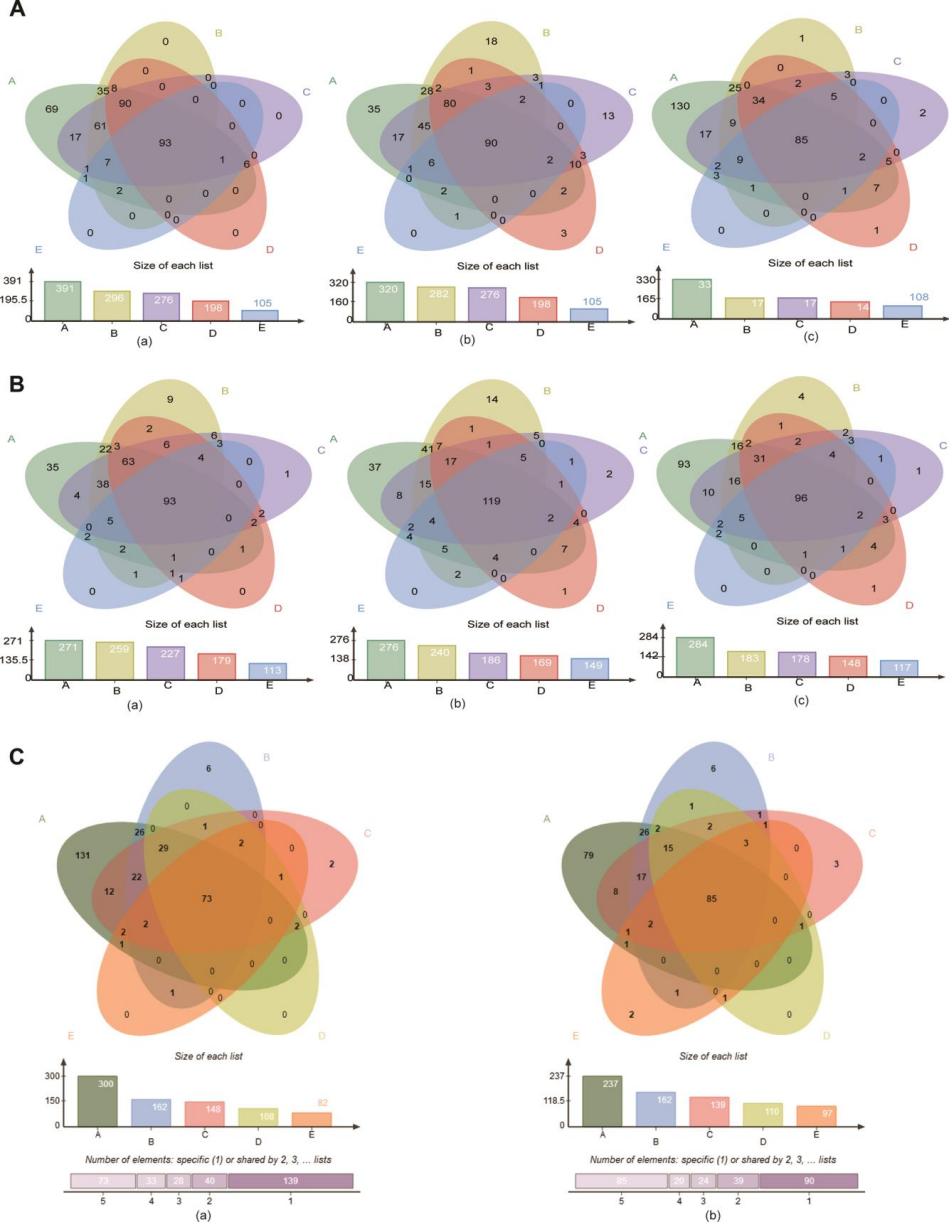
**The “core microbiome” of the age groups in the three sites.** (**A**) The “core microbiome” of various age groups at the genus level. (**a**) GCF; (**b**) SAL; (**c**) TB. (**B**) The “core microbiome” of various age groups at the species level. (**a**) GCF; (**b**) SAL; (**c**) TB. (**C**) The “core microbiome” of the three oral cavity sites with age at the genus level. (**a**), genus level; (**b**) species level.

Next, the “core microbiome” of the five age groups within each site, i.e., bacteria shared by all the age groups, was obtained by Venn analysis at the genus and species levels. At the genus level, 93, 90 and 85 genera were included in the “core microbiome” of GCF, SAL and TB, respectively (Figure 4A). At the species level, the number changed to 93, 119 and 96, respectively (Figure 4B). In all three sites, the number of genus and species contained in the “core microbiome” all decreased gradually with age (Figure 4A, 4B). The cross-site “core microbiome,” i.e., the bacteria shared by the GCF, SAL and TB within each age group, was then obtained by Venn analysis. At the genus level, the cross-site “core microbiome” consisted of 300, 162, 148, 108 and 82 genera for Groups A-E, respectively (Supplementary Figure 15). At the species level, the number was 237, 162, 139, 110 and 97, respectively (Supplementary Figure 15). The number of genera and species contained in the cross-site “core microbiome” decreased with age, and 73 genera and 85 species were shared by all age groups (Figure 4C). The results above suggest that the intra- and intersite “core microbiome” reduced their bacterial diversity significantly in aging mouth, which is consistent with the results of α-diversity.

### Correlations of the age- and site-related bacteria

**Figure 5:**
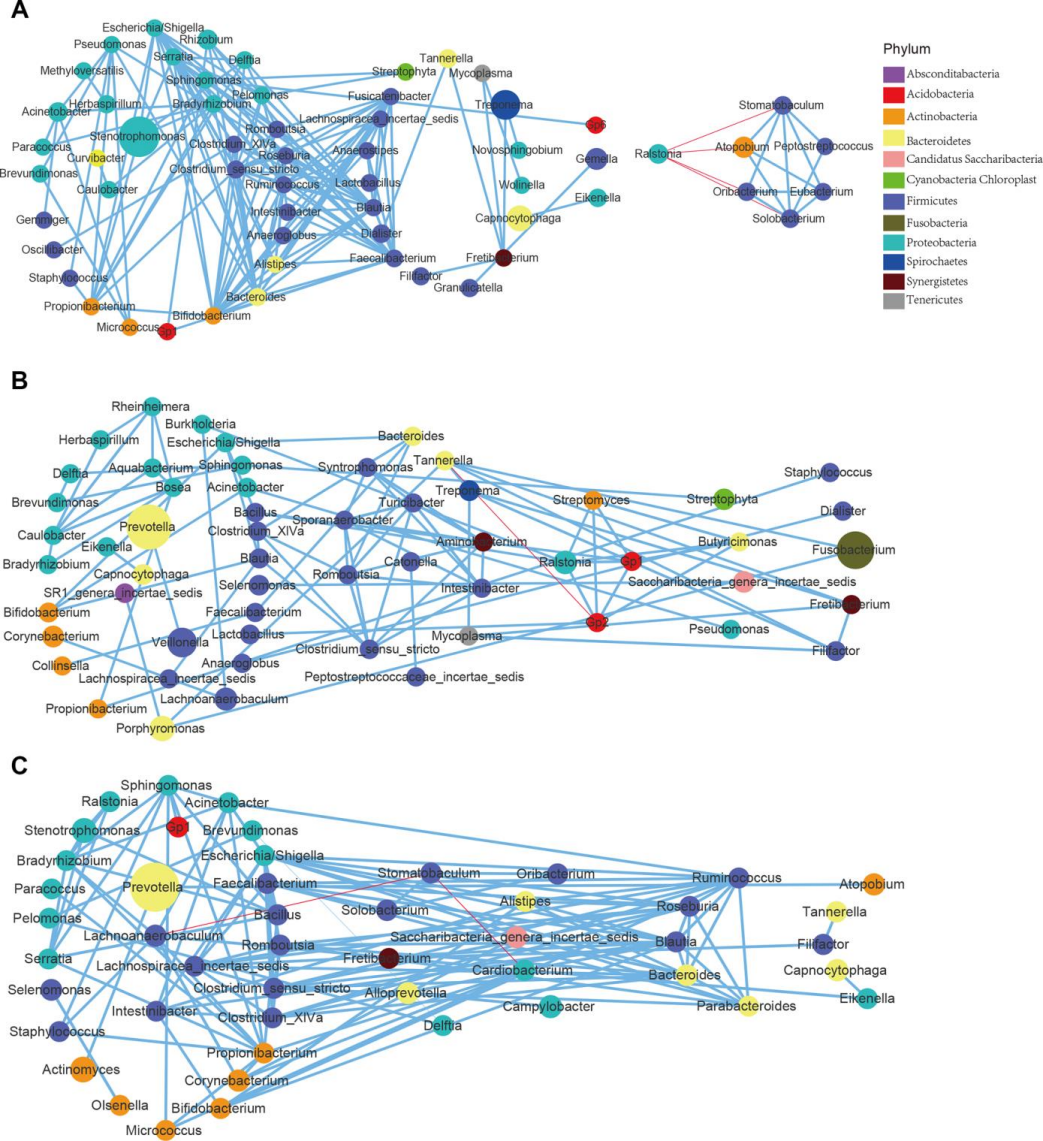
**Correlations of the differential bacteria differed among the age groups and across the sites.** (**A**) GCF; (**B**) SAL; (**C**) TB.

To study the correlation between the age-differentiated bacteria within each site, |Spearman correlation| ≥0.7 and q value≤0.01 were employed to analyze the prevalent taxa (≥0.02%) at the genus and species levels. The results showed that close correlations existed among the different bacteria in the age groups (Figure 5 and Supplementary Figure 16), and clear variations in the pattern of bacterial correlations were observed in each oral site, indicating the shift in bacterial correlation in response to aging and oral site variation (Figure 5 and Supplementary Figure 16).

### Using differential bacteria to distinguish age groups

**Figure 6:**
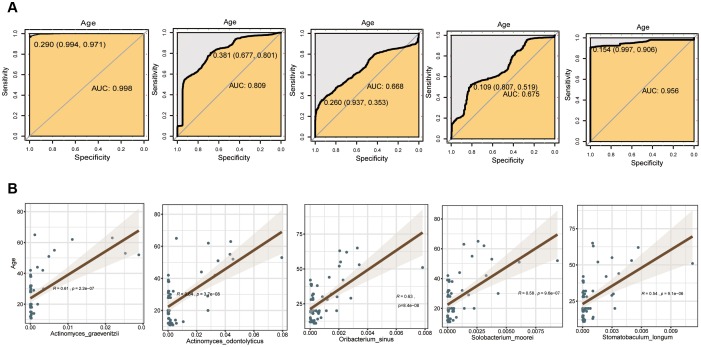
**Evaluation of the differential bacterial species of GCF with the random forest model.** (**A**) Accuracy rate of distinction. (**B**) Fitting bacteria species to age groups.

To explore the feasibility of using oral bacteria to assess mouth age or maturity, the random forest classifier model was employed for distinguishing different age groups. The data preparation of the random forest model was performed with 5-fold cross-validation, i.e., the original data was randomly divided into 5 sub-groups in each age group firstly; then, using a random sub-group as the test set, the remaining 4 sub-groups were used as training groups. The differential bacteria species with trends of GCF were first evaluated by the random forest model (Supplementary Figure 17), and the results showed that application of trending differential bacteria as a biomarker to distinguish Group A, B, C, D, and E could reach an accuracy of 0.998, 0.809, 0.668, 0.675 and 0.956, respectively (Figure 6A). The SAL and TB sites had many fewer different bacteria species than GCF, so the accuracy rates were relatively lower; the value was 0.804, 0.677, 0.835, 0.642 and 0.620 for Group A-E for SAL, respectively; and 0.831, 0.597, 0.515, 0.663 and 0.631 for Group A-E for TB, respectively (Supplementary Figures 18, 19). Meanwhile, *Actinomyces odontolyticus, Oribacterium sinus* and *Solobacterium moorei* were used for linear fitting of the age groups B, C, and D, and much smaller p-values were obtained (Figure 6B).

## DISCUSSION

The microbiota coexists with humans as an ecosystem and reflects the health or disease state of the host, playing an important role in human health and disease [18, 19]. Some host factors, such as immunity, genetics, or different anatomical states of a body site, may also affect the composition and stability of the microbiota [20, 21]. Therefore, revealing the characteristics of the microbiota with age may be the key to exploring age-related diseases such as oral diseases and type 2 diabetes. Determining the variation pattern of the bacterial composition with age is a prerequisite for studying the characteristics of microbiota and developing potential applications. However, the dynamic changes in the microbiome of individual oral sites over a lifetime have not been well characterized. In this study, we systematically studied the changes in oral flora with aging. Our research results revealed that the α-diversity of bacteria has a tendency to decrease over time while the β-diversity has an increasing trend. Moreover, the microbial composition analysis indicated the variation in the patterns of bacterial composition over time and across oral sites, as well as a bell-shaped trend of intersite differential bacteria in aging mouth. The subsequent intra- and intersite “core microbiome” results showed a significant reduction in the bacterial diversity over time, which is consistent with the α-diversity results. Next, the correlation investigation indicated the interdependence of bacteria in the oral microbiota, the change in their correlation with age, and the different patterns of correlation between sites at the genus and species levels. Last, application of the differential bacteria as a biomarker to distinguish groups reached a high accuracy in some oral sites.

There are a variety of unique microenvironments in the mouth, such as hard teeth, non-shedding surfaces and mucosal epithelial surfaces. These surfaces are exposed to the fluid phase of the saliva or to the gingival crevicular fluid (GCF). The microbial communities that inhabit on these surfaces are also different [22, 23]. Saliva forms a film approximately 0.1 mm deep across the inner surface of the mouth and plays an important role in maintaining dental health by washing away microorganisms and neutralizing the acid produced by bacteria. In this study, the intersite comparison of bacterial diversities, microbial composition, and bacterial correlation indicated the distinction between sites in response to aging. The reason for the differentiation can be attributed to the environmental factors such as epithelial cells exposed to the flora, AMPs, mucus barriers, SIgAs, epithelial microvilli, epithelial tight junctions, epithelial metabolism, oxygen barriers, and miRNA, etc. However, other factors also cannot be ignored: changes in metabolism and daily activities. For example, each age group has its own metabolic characteristics: young people have a high level of metabolism while the metabolism of the elderly is slower. Young and middle-aged people have a high frequency of social activities, i.e., they will travel to a variety of different places, and this is relatively less likely to occur for teenagers and the elderly. All of these factors have shaping effects on microbiota and trigger a change in the microbial composition over time. Our research confirmed this hypothesis; the number of intersite differential bacteria showed a trend of increasing at first and then decreasing (bell-shaped) with age, and the bacterial correlation was relatively higher among the younger groups.

Changes in the “core microbiome” represent functional variation; it affects the health of the body and the occurrence of diseases in turn. In the present study, we observed a significant reduction in the bacteria of the “core microbiome” in all three oral sites; this indicates that oral bacteria develop dysbiosis with age. The bell-shaped trend of intersite differential bacteria with age further validated the occurrence of dysbiosis in oral cavity. It is worthwhile to conduct intensive research to study the biological implications in the future. Because the oral cavity is exposed to the external environment and is subject interference by brushing and eating every day, the microbes in saliva and the fluid associated with teeth and tongue may change significantly, which will affect their usage for risk assessment, diagnosis or prognosis. However, we found that the “core microbiome” sustained a considerable number of bacteria despite the floral shift, and these bacteria may be associated with oral health and basic functions. On the other hand, we discovered that the species and abundance of bacteria vary with age. The study by Socransky et al. revealed disease associations of specific bacterial organisms, including Porphyromonas, Treponema, and Tannerella, which were classified as the red complex organisms [24], and we did find that these bacteria increase with age in modules with definite trends in SAL and TB sites, which could be a reason why the elderly are prone to oral diseases. Moreover, some health-related bacteria such as *Feacalibacterium prausnitzii* were also found to have marked variation with age. More focus should be placed on the variable microbiota in the future and to elucidate its contribution to health maintenance and transition into disease.

Microbial communities are bound to affect health, and a better understanding of their dynamic complexity may contribute to the development of diagnostic medical tools. Ideally, personal health can be optimized by manipulating microbial communities, which can be helpful to develop more specific treatments. We systematically studied the changes in oral flora with age and found that the oral flora changed significantly with age; this might be another reason for the high incidence of diseases, especially oral diseases, with age in addition to the host's own genetic and metabolic factors, providing new ideas for the etiology of diseases.

## MATERIALS AND METHODS

### DNA extraction, library construction and sequencing

### Extraction of DNA

Total DNA was extracted with CTAB/SDS method. 1% agarose gels was used for checking the DNA concentration and purity. DNA was diluted to 1ng/μl using sterile water based on the obtained concentration.

### Library construction and sequencing

Genome DNA from all the samples was used as amplification templates. PCR primers were from the V3-V4 region of 16S rDNA, forward primer, 5'-ACTCCTACGGGAGGCAGCA-3'; and reverse primer, 5'-GGACTACHVGGGTWTCTAAT-3'. All PCR reactions were carried out in 30μL total volume with 15μL of Phusion® High-Fidelity PCR Master Mix (New England Biolabs), 0.2μM of forward and reverse primers, and about 10ng templates DNA. Thermal cycling included an initial denaturation step at 95°C for 5 min, followed by 30 cycles of denaturation at 95°C for 30s, annealing at 50°C for 30s, and elongation at 72°C for 40s. Finally 72°C for 7 min.

Then the PCR products were purified with GeneJET Gel Extraction Kit (Thermo Scientific) and qualified by electrophoresis on 2% agarose gel, samples with single amplification product were chosen for further experiments. The library was sequenced on an Illumina Hiseq 2500 platform at Novogene Company (Beijing, China).

### Data processing

The quality of the Raw sequence was filtered and analyzed using the next-generation microbiome bioinformatics platform (QIIME2 version 2018.6 pipeline) for data preprocessing. A software package included with Usearch was used to identify the exact sequence variants (ESV). α- and β-diversity analyses were performed using the phyloseq software in R package. α-diversity was calculated by Faith's Phylogenetic Diversity, Shannon index, and observed OTUs. Principal coordinate analysis (PCoA) was analyzed based on the Bray_Curtis distance, Bray-Curtis variability is an indicator used to measure differences in taxonomic composition in ecology. PCoA is a method to explore and to visualize similarities or dissimilarities of data. It sorts the data through a series of eigenvalues and eigenvectors, selects the main eigenvalues in the first few places, finds the most important coordinates in the distance matrix, and perform a rotation on the data matrix. PCoA does not change the mutual positional relationship between the sample, but only changes the coordinate system. PCA is based on the sample similarity matrix (such as Euclidean distance) to find principal components, while PCoA is based on dissimilar distance matrix (other distances than Euclidean distance, including binary_jaccard, bray_curtis, unweighted_unifrac, and weighted_unifrac distance) to find principal coordinates. Statistical differences in β-diversity were analyzed using permutation-arranged multivariate analysis (PERMANOVA, R function Adonis (vegan, 999 permutations)). Usearch was used to cluster the Effective Tags of all samples, and the sequences cluster into 372 operational taxon units (OTUs) with 97% identity, and then perform representative sequences of OTUs according to the database Ribosomal Database Project (The species annotation is RDP version 11.5). At the genus and species levels, the differences between age groups were analyzed. The “pheatmap” for the heat map, and the average relative abundance in the group were displayed with a histogram (R package: “ggplot2”). Correlation analysis was performed at the genus and species levels, all the differential genera and species identified in the variation analysis were pooled for spearman correlation analysis of their correlation with all the bacteria and clinical parameters after 0.002% and 30% screening (R package: “Hmisc”). The genera and Species with padj<=0.05, |cor|>=0.5 were shown with a network map (software cytoscape, R package “pheatmap”). To further explore the relationship between oral microbiota and age, we used a random forest algorithm to predict the classification of samples. Random forest regression was performed with 1,000 regression trees based on 5-fold cross-validation, 80% of the samples were randomly selected for model training and the remaining 20% were used for validation. The predicted result is displayed by the roc curve (R package: “proc”). In the random forest prediction classification algorithm, the contribution of different species can be known. The top species were selected and the random forest algorithm prediction classification was performed.

### Ethics statement and samples collection

All saliva samples used in this study were collected from the Stomatological Hospital of Shandong University (Jinan, Shandong). Please refer to Supplementary Table 1 for more information of the sampling patients. This study was reviewed and approved by the Ethics Committee of the Stomatological Hospital of Shandong University. Informed consent was signed by all volunteers prior to inclusion in the study. All methods are implemented in accordance with the relevant guidelines and regulations.

Saliva specimens were collected 2 hours after a meal: mouth was rinsed with sterile double distilled water for 3 times firstly before collecting saliva, then the saliva samples were collected with 1.5μL centrifuge tubes. To collect a gingival crevicular fluid sample, 2 paper points (Henry Schein, Almere, Netherlands) were inserted to the bottom of the gingival pocket for 10 s afer supragingival plaque removal, and the paper points after sampling were immersed in sterile phosphate buffer saline. For tongue back sample, a cotton swab was use to collect fluid at the soft tissue of the tongue back, and the cotton swab after sampling were immersed in sterile phosphate buffer saline. All samples were immediately transferred to the laboratory within 20 minutes and stored at -80 °C for later usage.

### Availability of data

The sequencing data will uploaded to the NCBI website with a BioProject ID PRJNA number and available at https://www.ncbi.nlm.nih.gov/sra/PRJNA.

## Supplementary Material

Supplementary Figures

Supplementary Tables 1, 4, 5

Supplementary Table 2

Supplementary Table 3

Supplementary Table 6
