# Supplementary material for: Microbiome succession with increasing age in three oral sites
Source: Aging (Albany NY). 2020 May 7;12(9):7874–907. doi: 10.18632/aging.103108 (PMC7244077; doi:10.18632/aging.103108)
Supplement: Supplementary Figures [file aging-12-103108-s005..pdf]

## SUPPLEMENTARY FIGURES

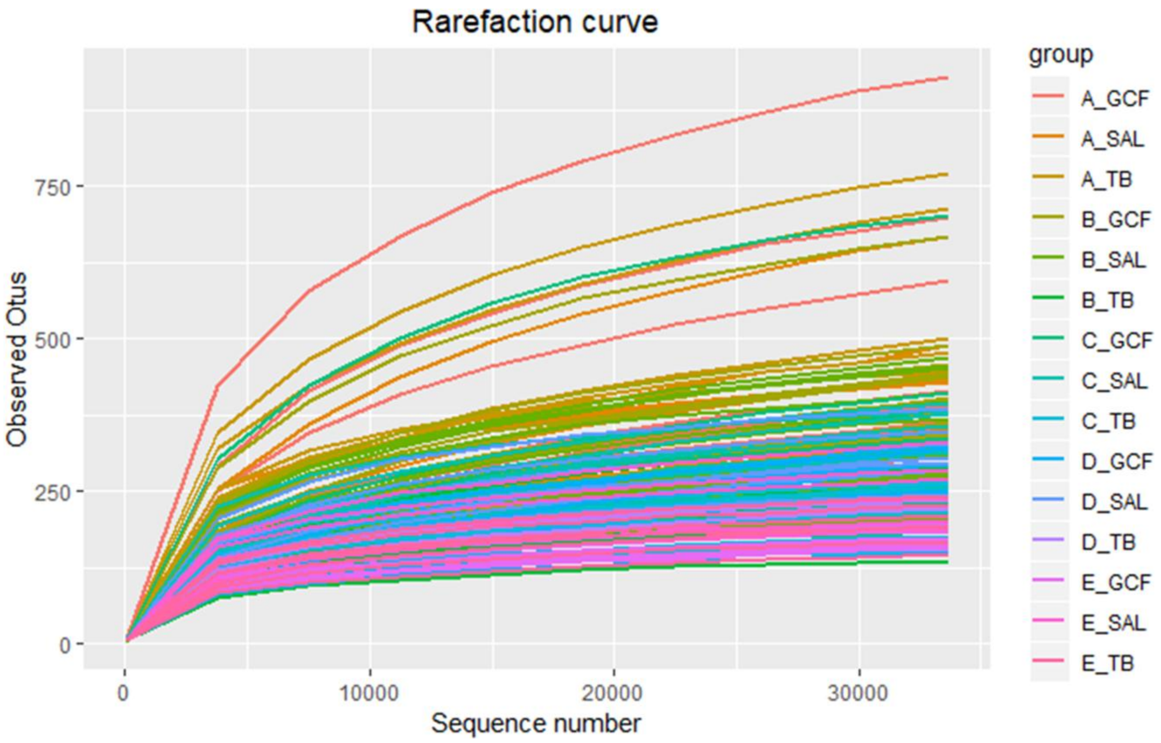


**Supplementary Figure 1. Rarefaction curve: the abscissa is the number of randomly selected sequences and the ordinate is the number of OTUs clustered based on the number of sequences.** Each curve represents a sample, and the curve tends to be stable, indicating that the number of extracted sequences is enough to reflect the OTUs of the sample.

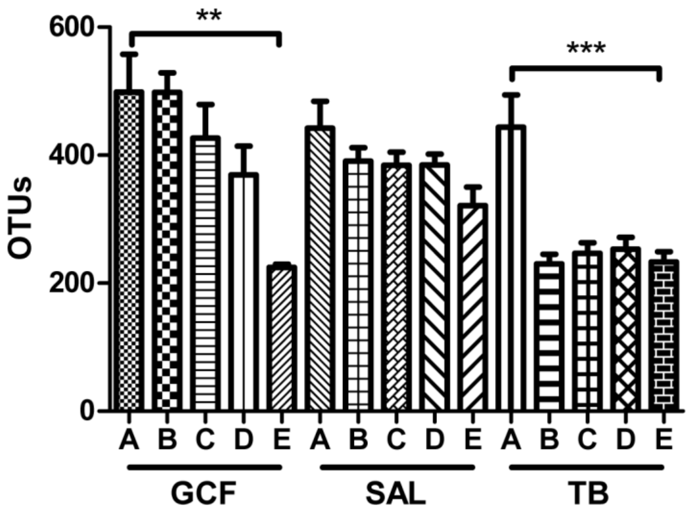


**Supplementary Figure 2. OTUs of the samples.**

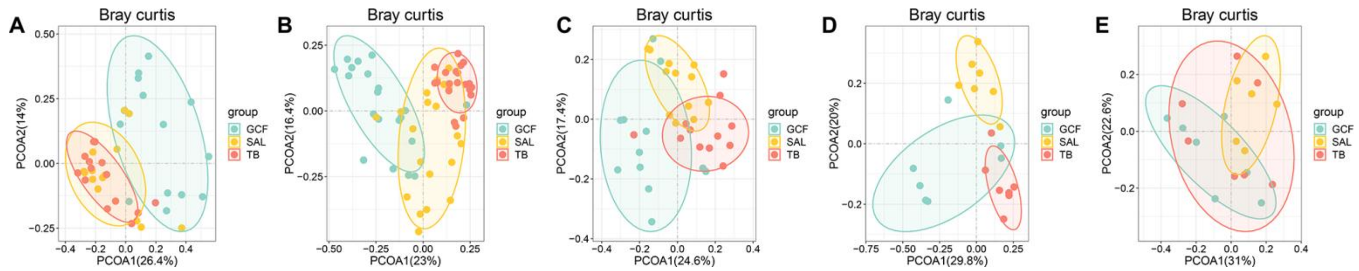


**Supplementary Figure 3. The β-diversity indexes of bacteria over time differentiated in the three oral sites.** (**A**–**E**) were age groups.

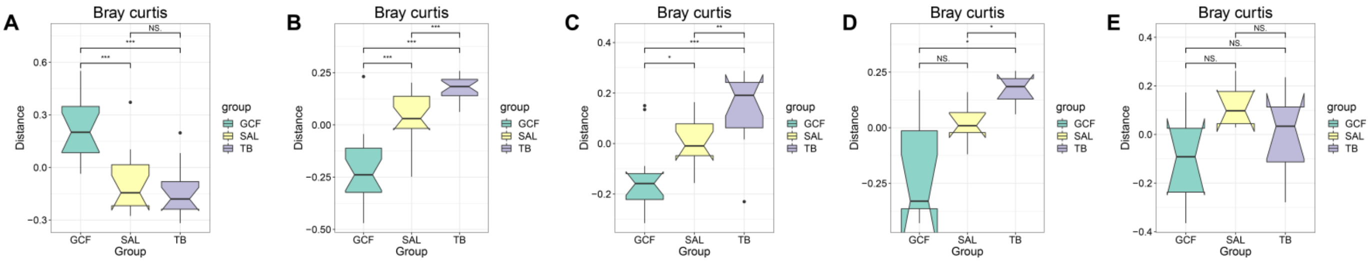


**Supplementary Figure 4. The first principal component analysis of microbial changes with age in various oral sites.** (**A**–**E**) were age groups.

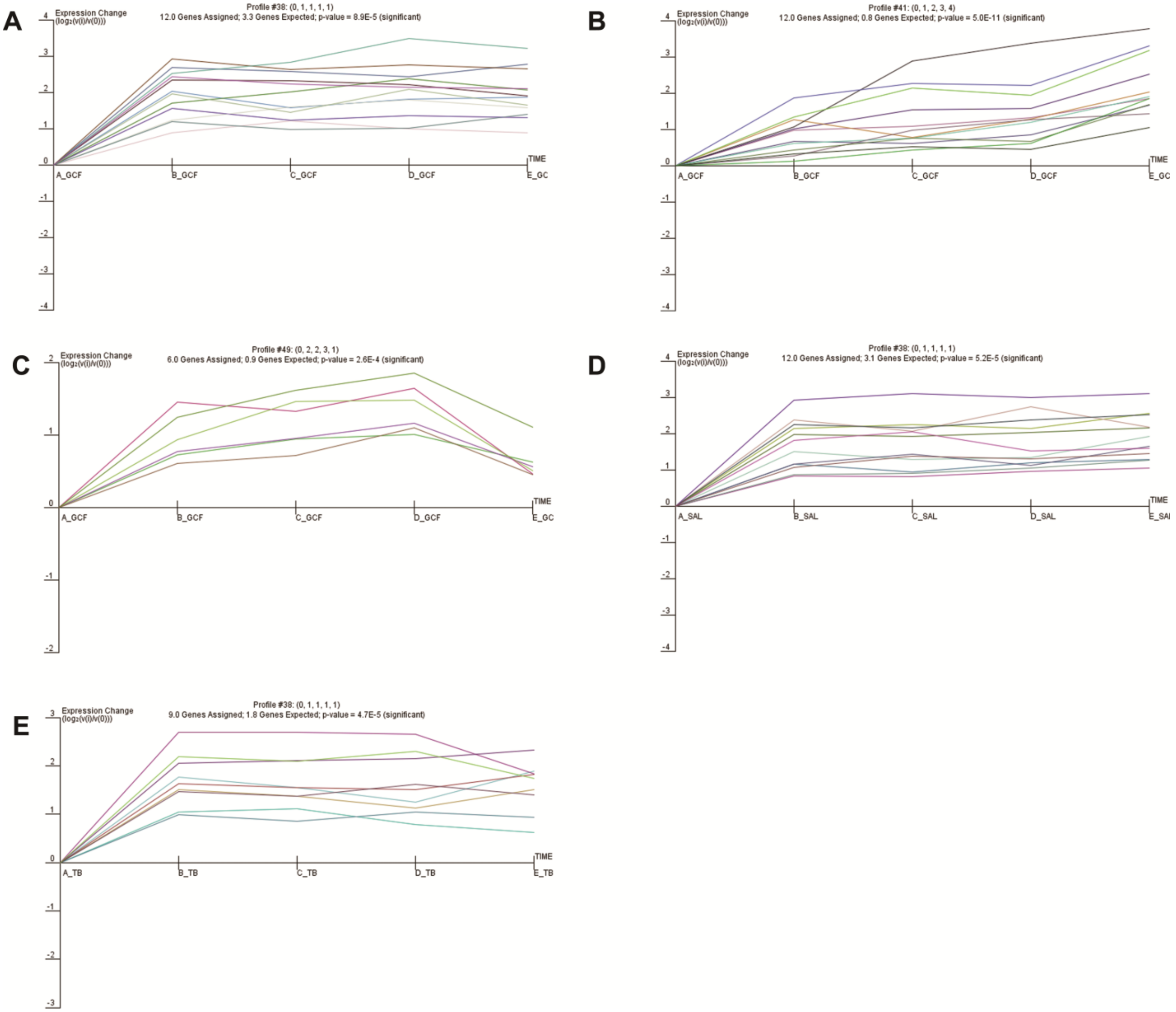


**Supplementary Figure 5.The bacteria contained in the module changed over time.** (**A**–**C**) were GCF moduals; (**D**), SAL module; (**E**), TB module.

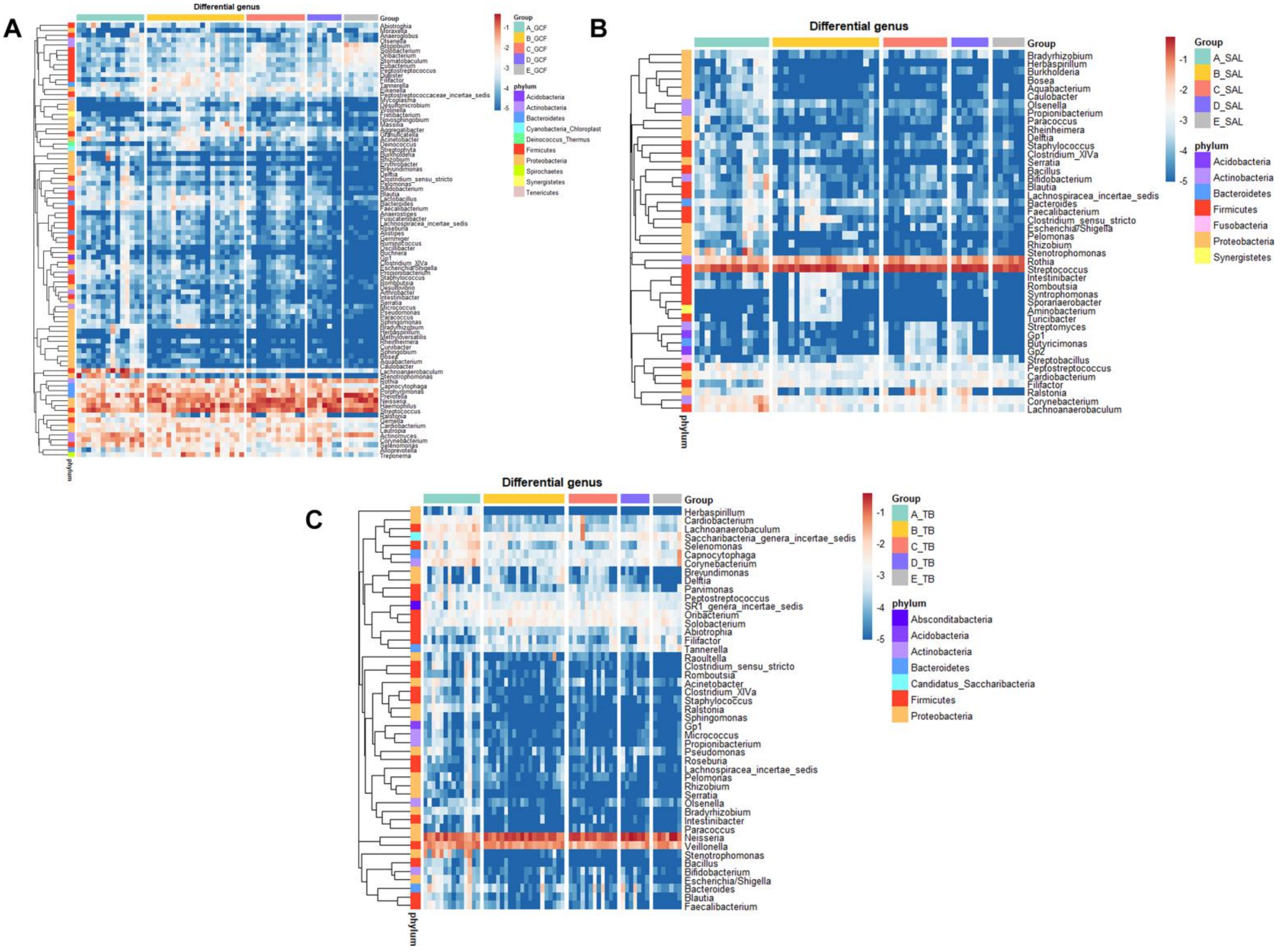


**Supplementary Figure 6. The genus that had marked variation with age.** (**A**) GCF; (**B**) SAL; (**C**) TB.

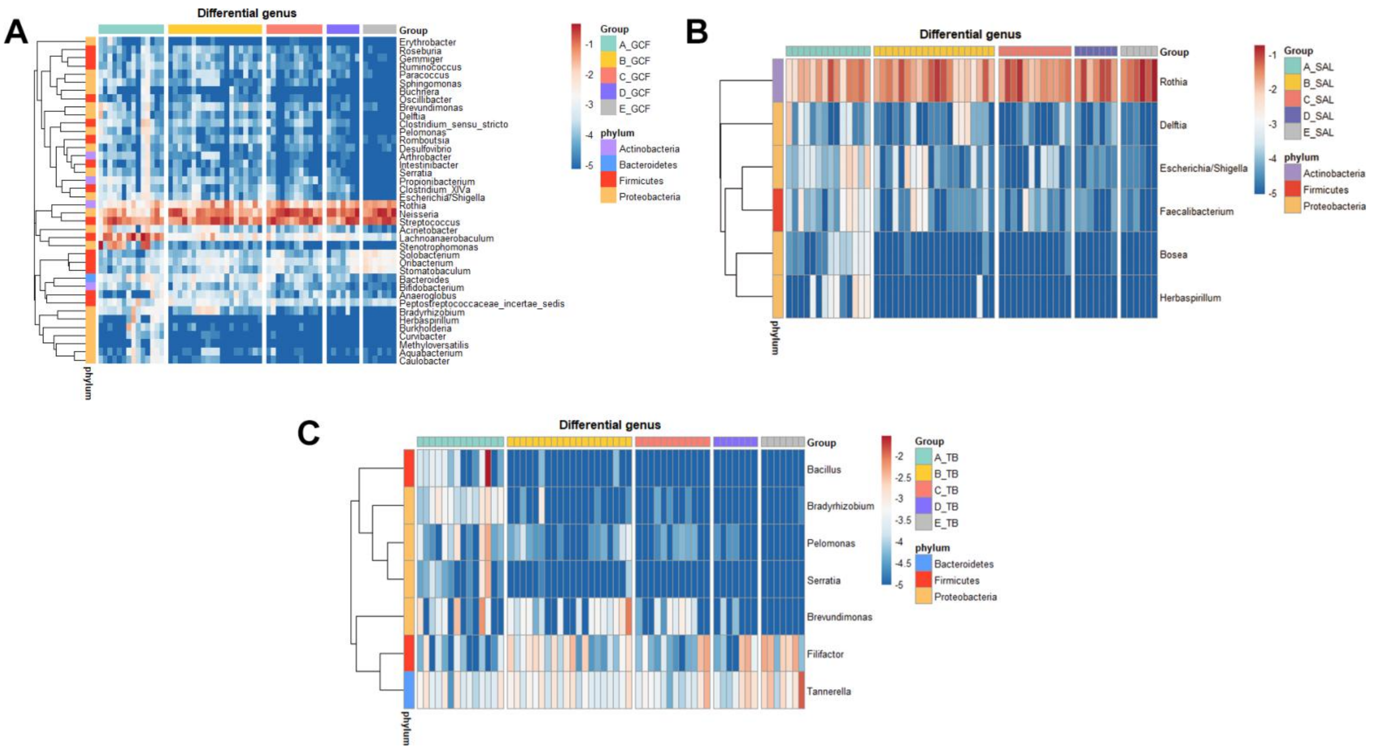


**Supplementary Figure 7. The genus that underwent gradual changes with age.** (**A**) GCF; (**B**) SAL; (**C**) TB.

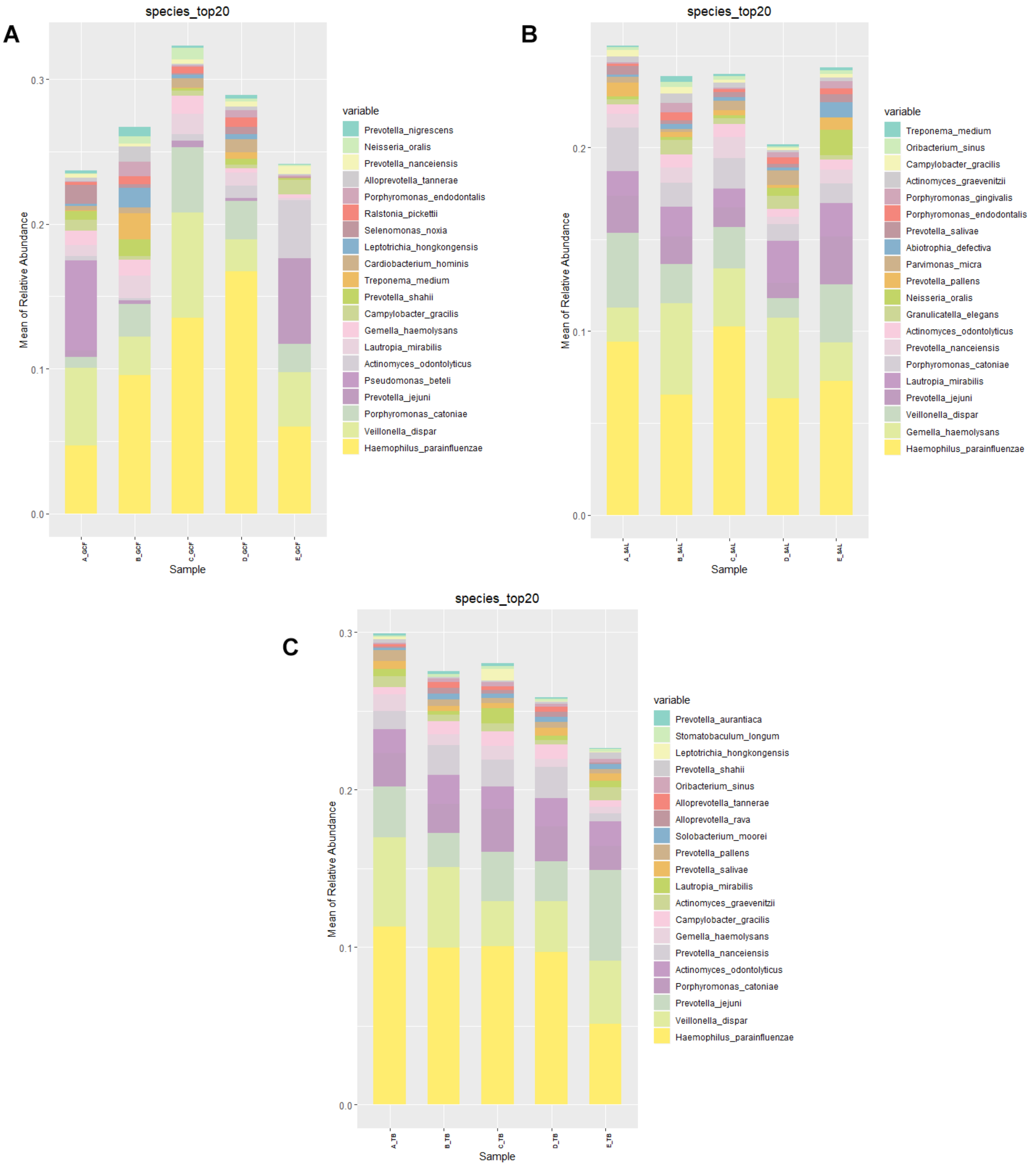


**Supplementary Figure 8. The composition of microbiota at the species level.** (**A**) GCF; (**B**) SAL; (**C**) TB.

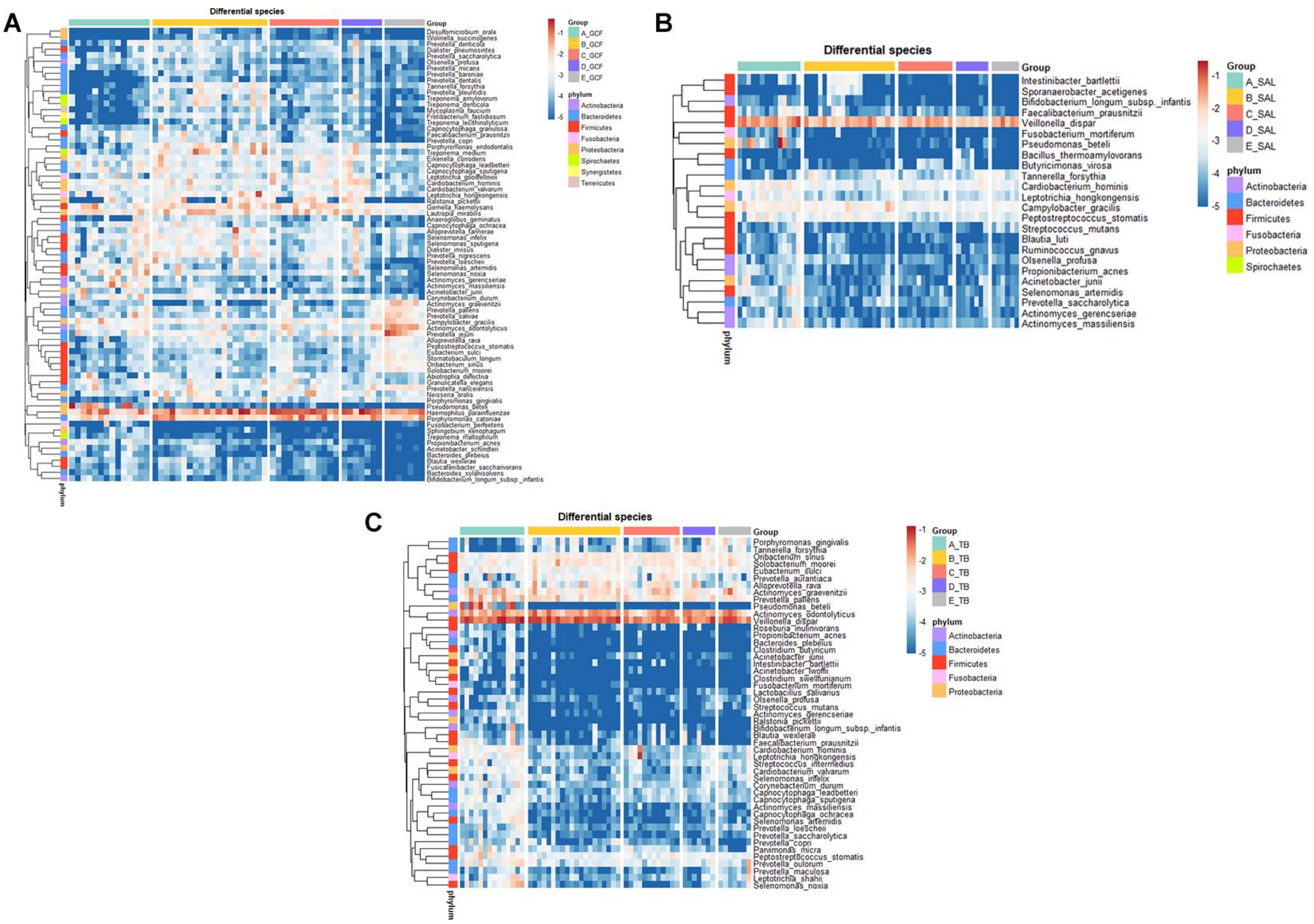


**Supplementary Figure 9. The species that underwent significant changes.** (**A**) GCF; (**B**) SAL; (**C**) TB.

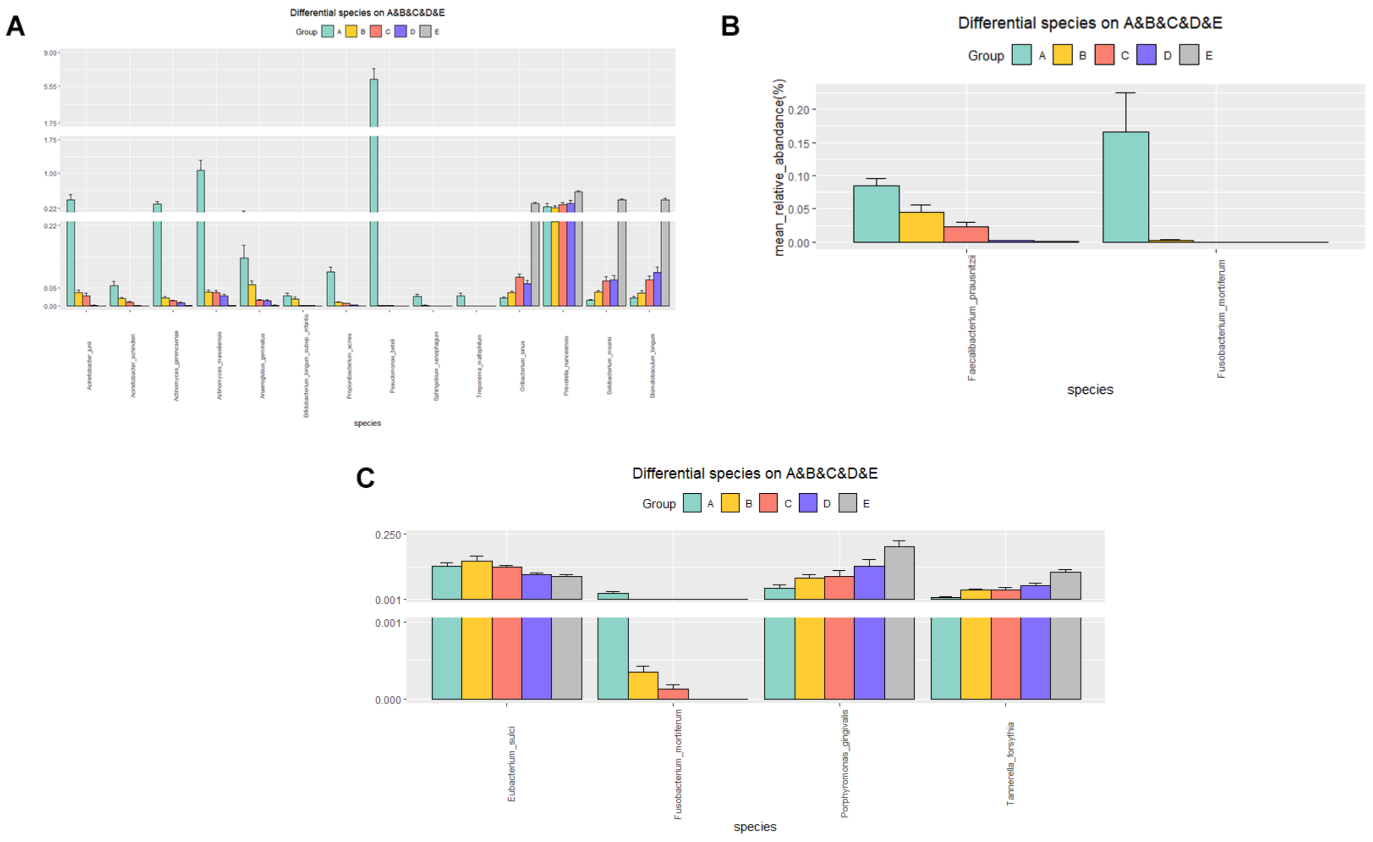


**Supplementary Figure 10. The species with gradual variations with trend.** (**A**) GCF; (**B**) SAL; (**C**) TB.

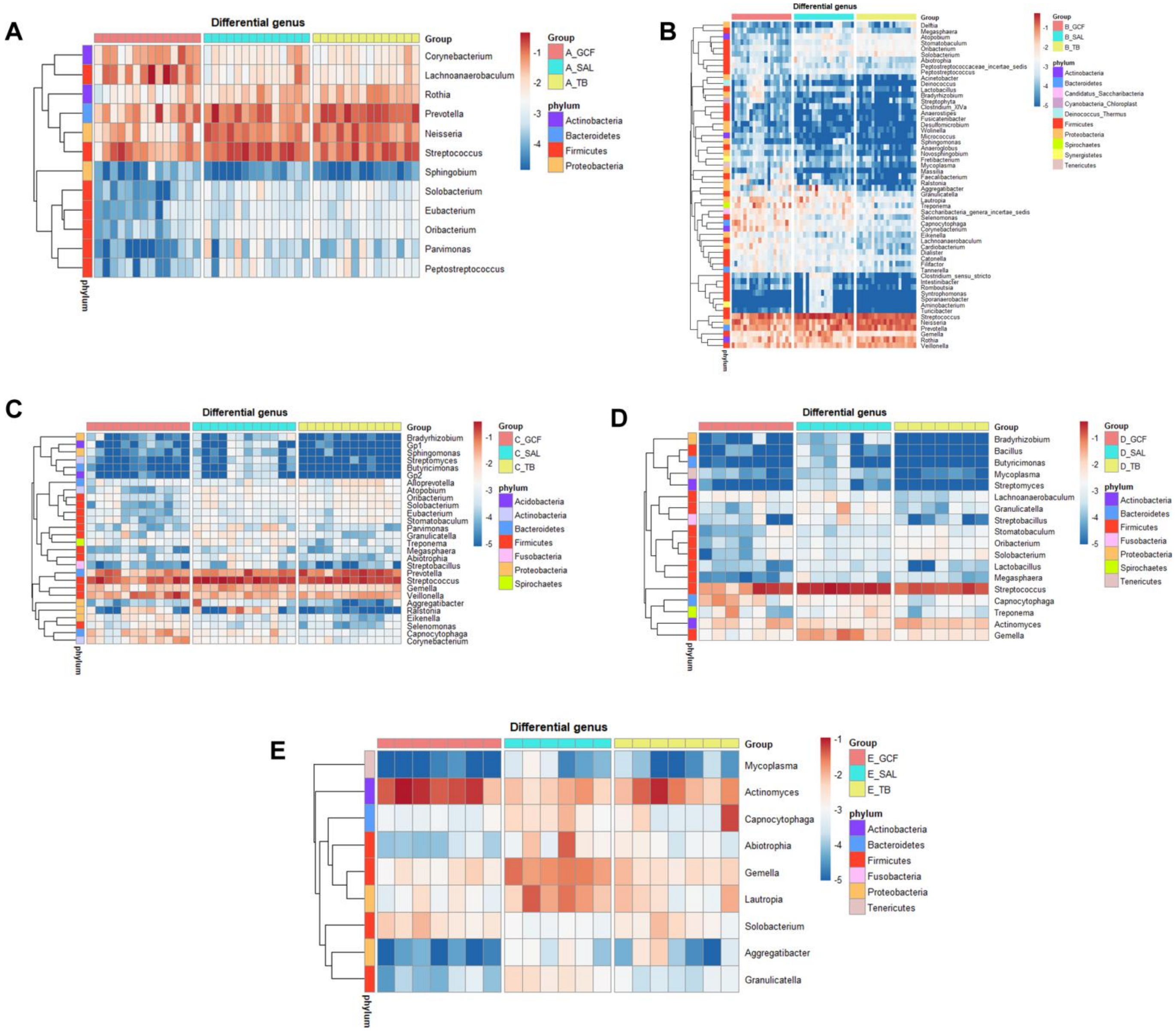


**Supplementary Figure 11. The genera differently distributed in the GCF, SAL and TB sites of oral cavity.** (**A**–**E**), age groups.

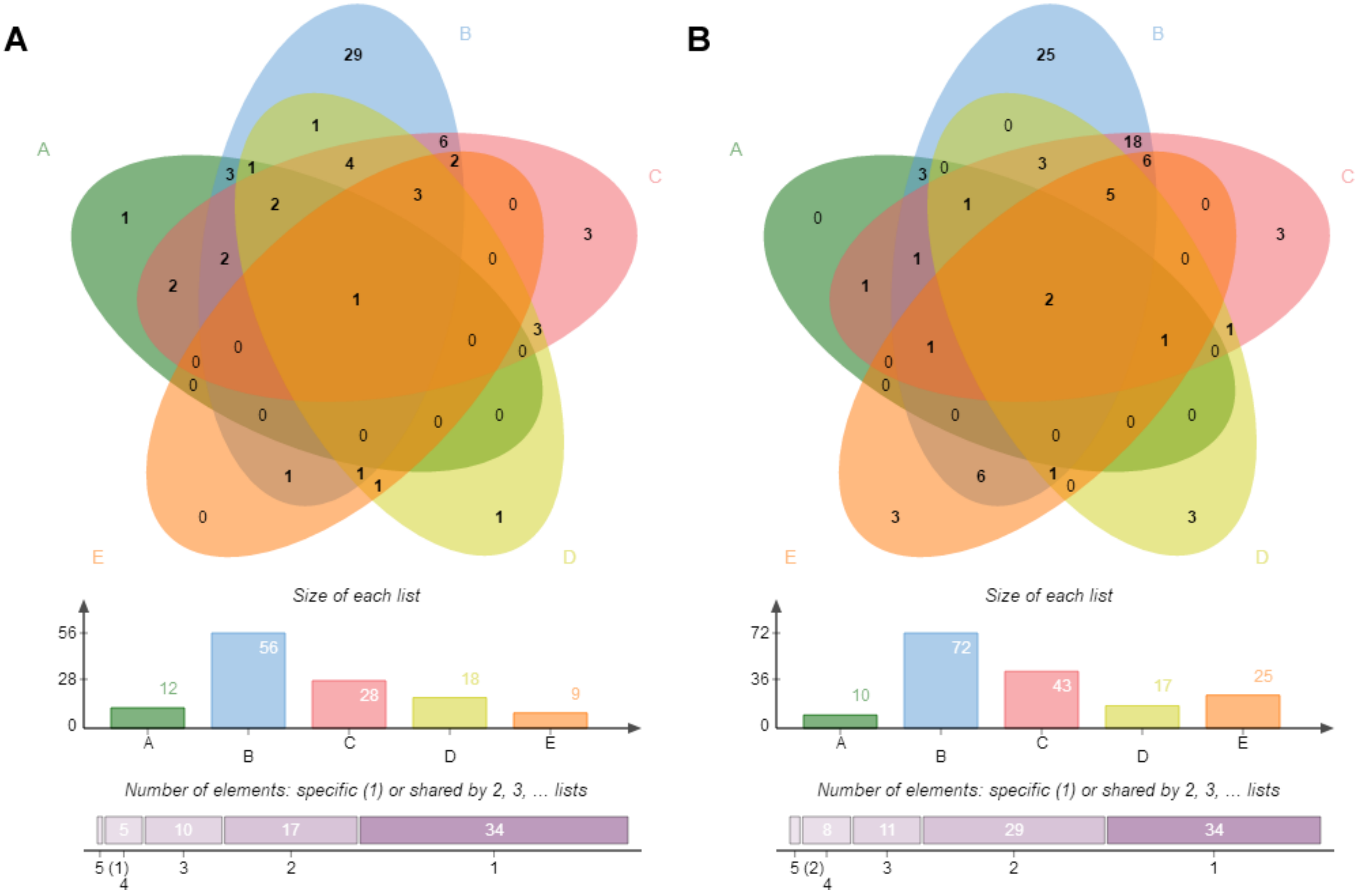

**Supplementary Figure 12. The differential genus and species shared by the three sites of oral cavity at various ages.** (**A**), at the genus level; (**B**), at the species level.

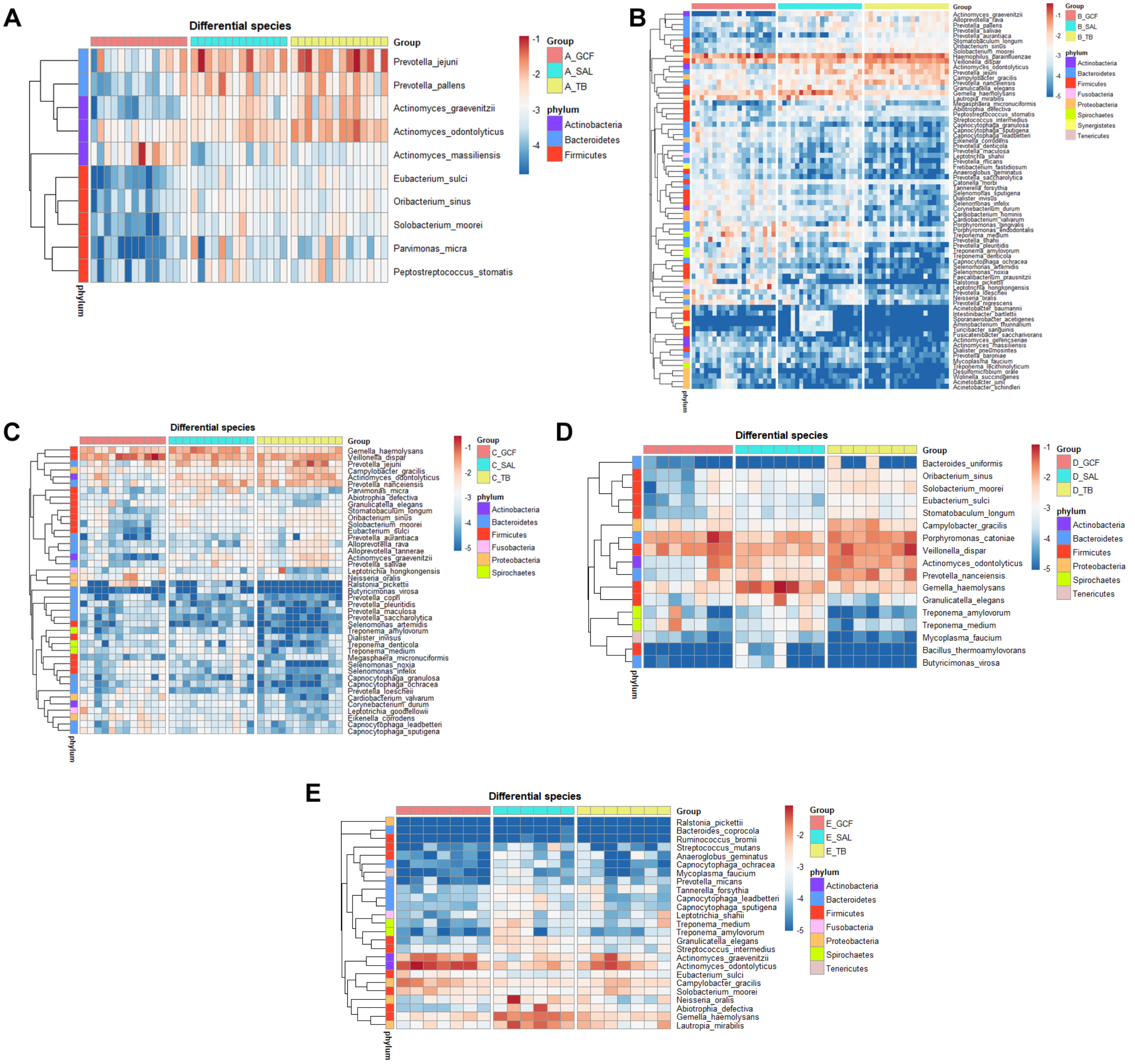


**Supplementary Figure 13. The species differently distributed among the GCF, SAL and TB sites of oral cavity.** (**A**–**E**), age groups.

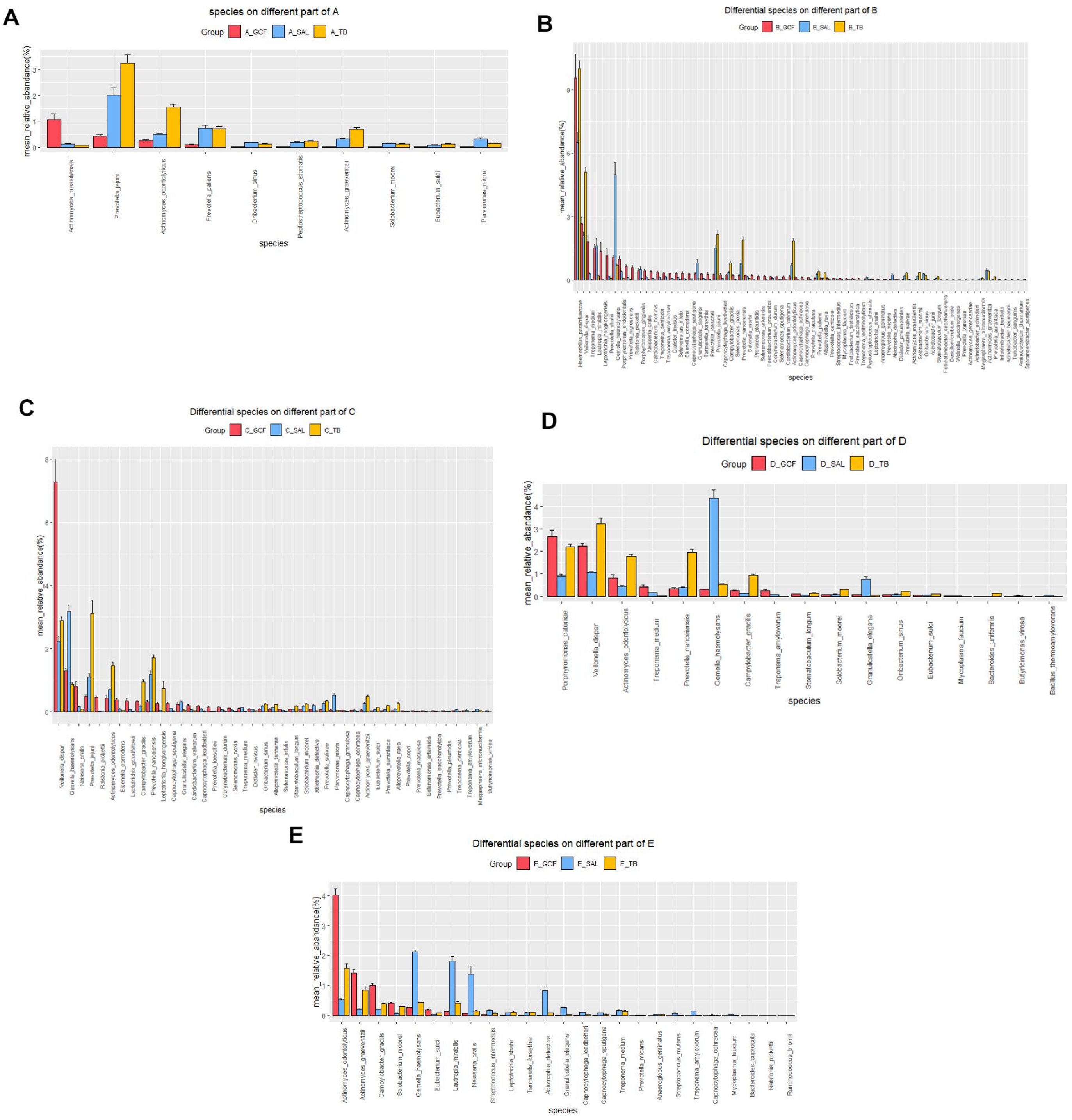


**Supplementary Figure 14. The species differently distributed among the GCF, SAL and TB sites of oral cavity in various age groups.** (**A**–**E**), age groups.

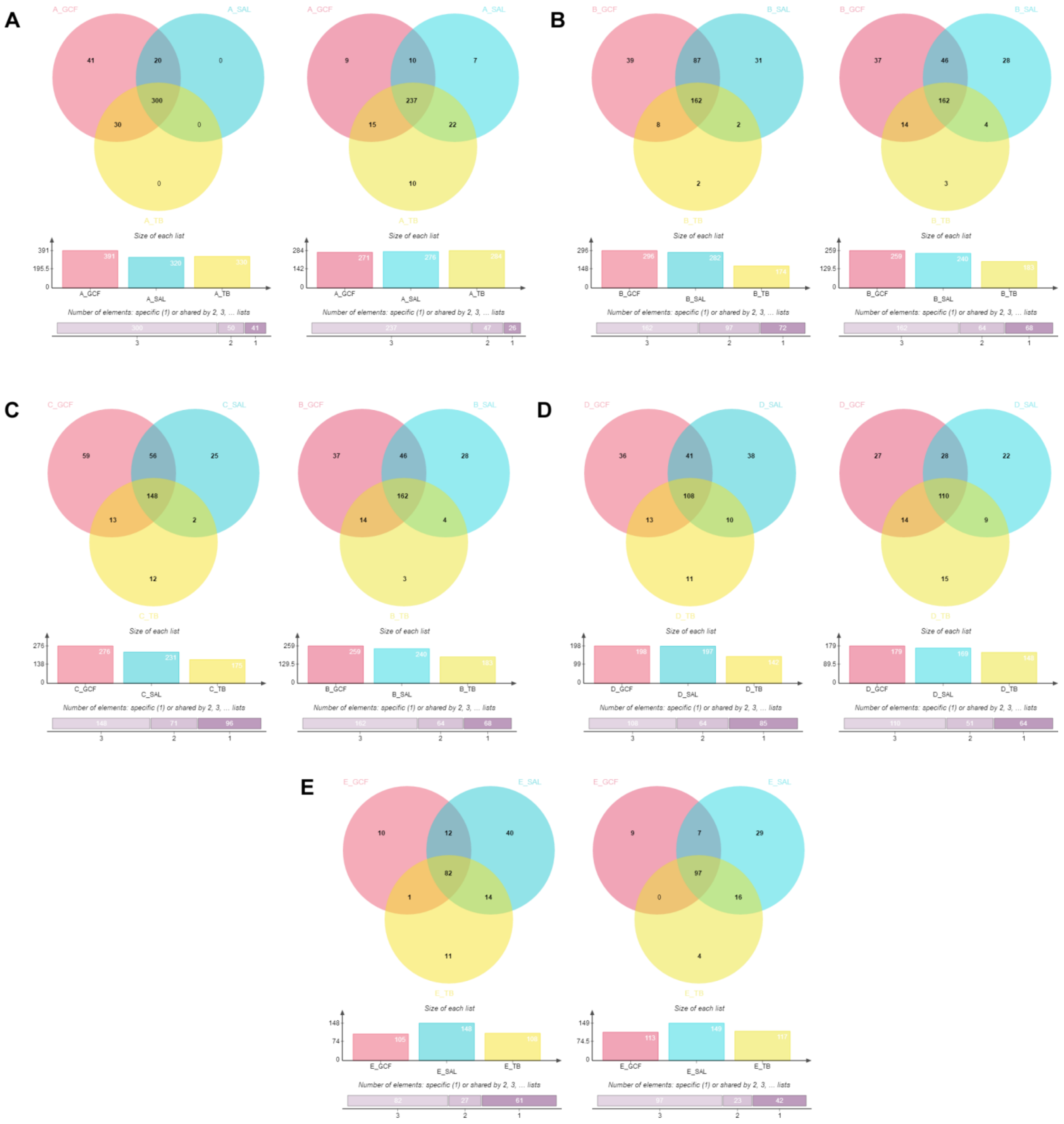


**Supplementary Figure 15. The "core microbiome" of the three sites of oral cavity with age at the genus and species levels.** (**A**–**E**), age groups.

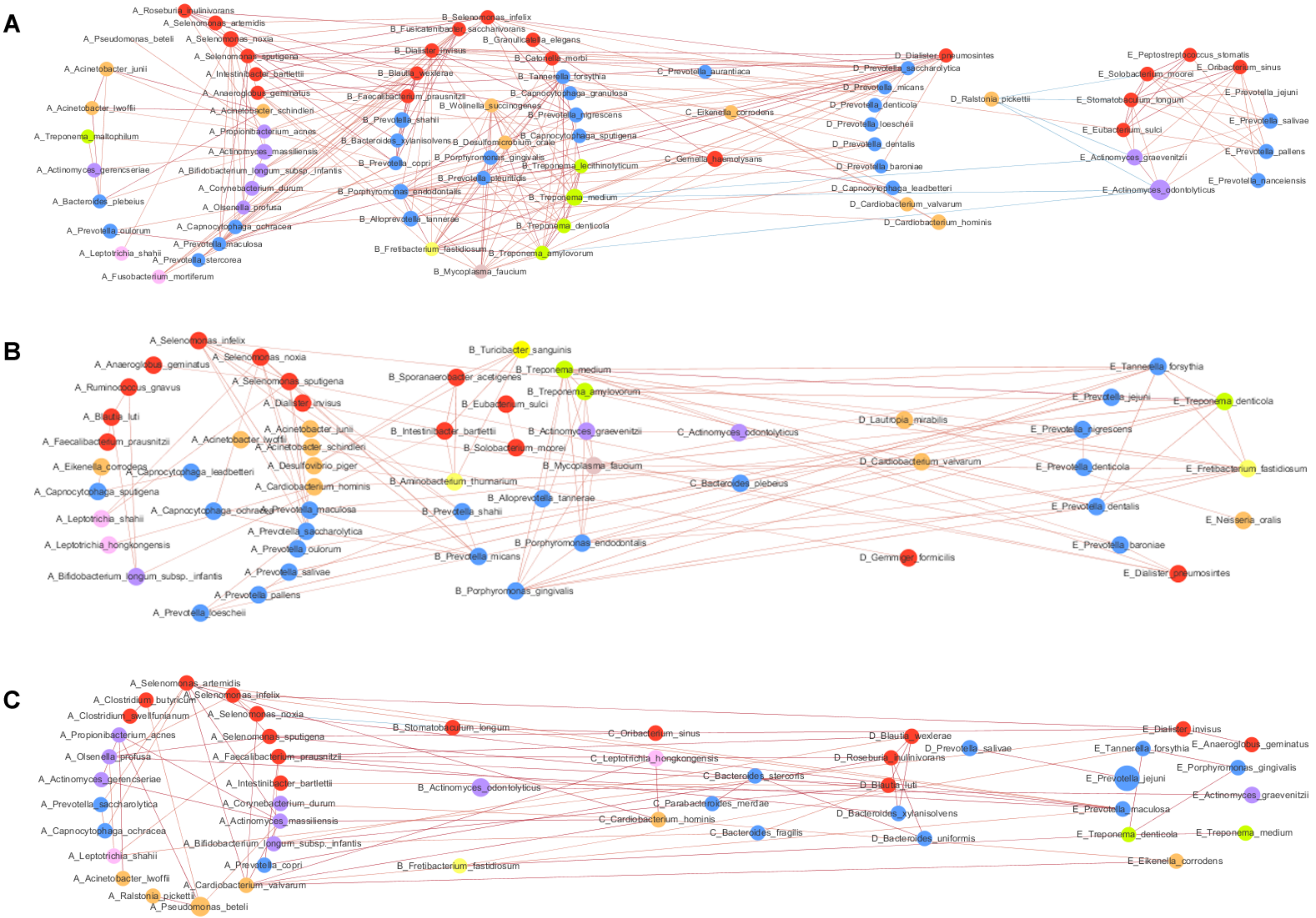


**Supplementary Figure 16. Correlation analysis of the oral microbiota at the species level.** (**A**) GCF; (**B**) SAL; (**C**) TB.

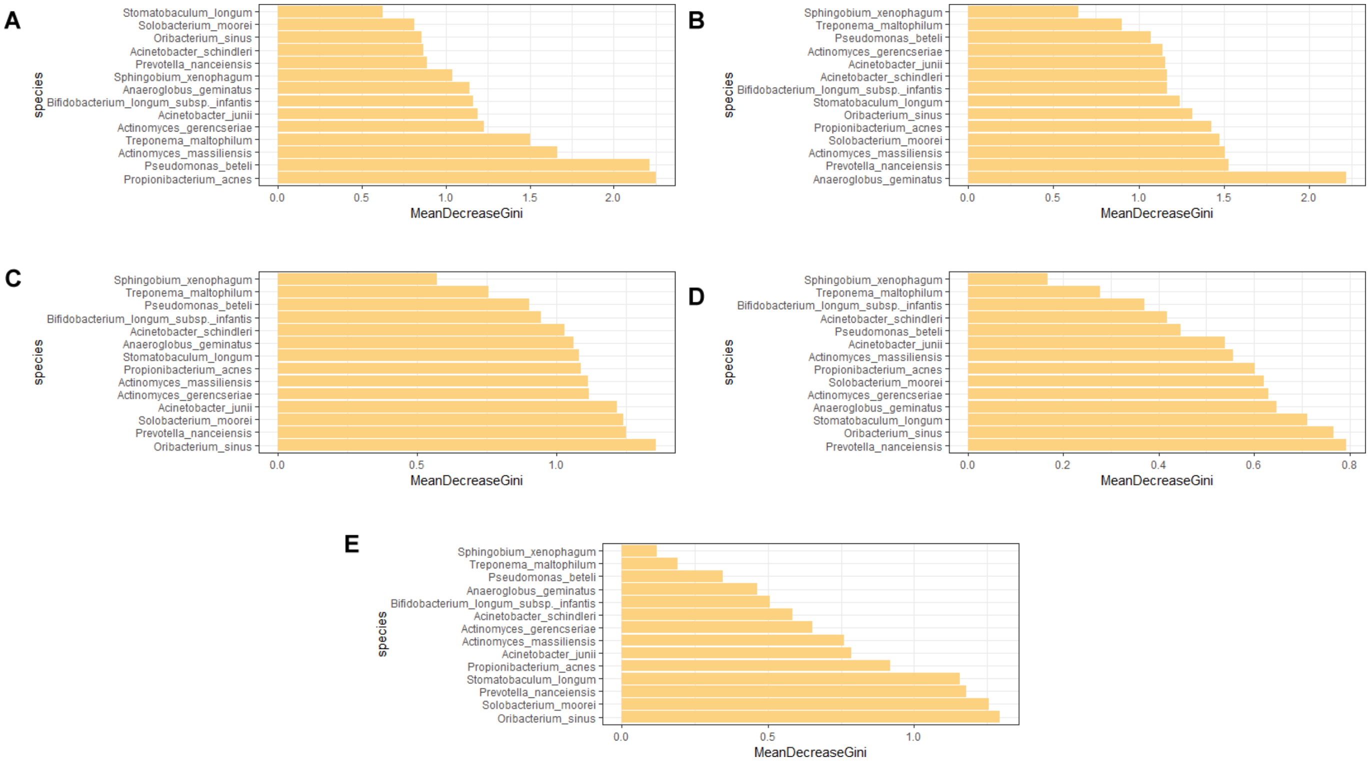


**Supplementary Figure 17. Evaluation of the differential bacterial species of GCF with the random forest model.**

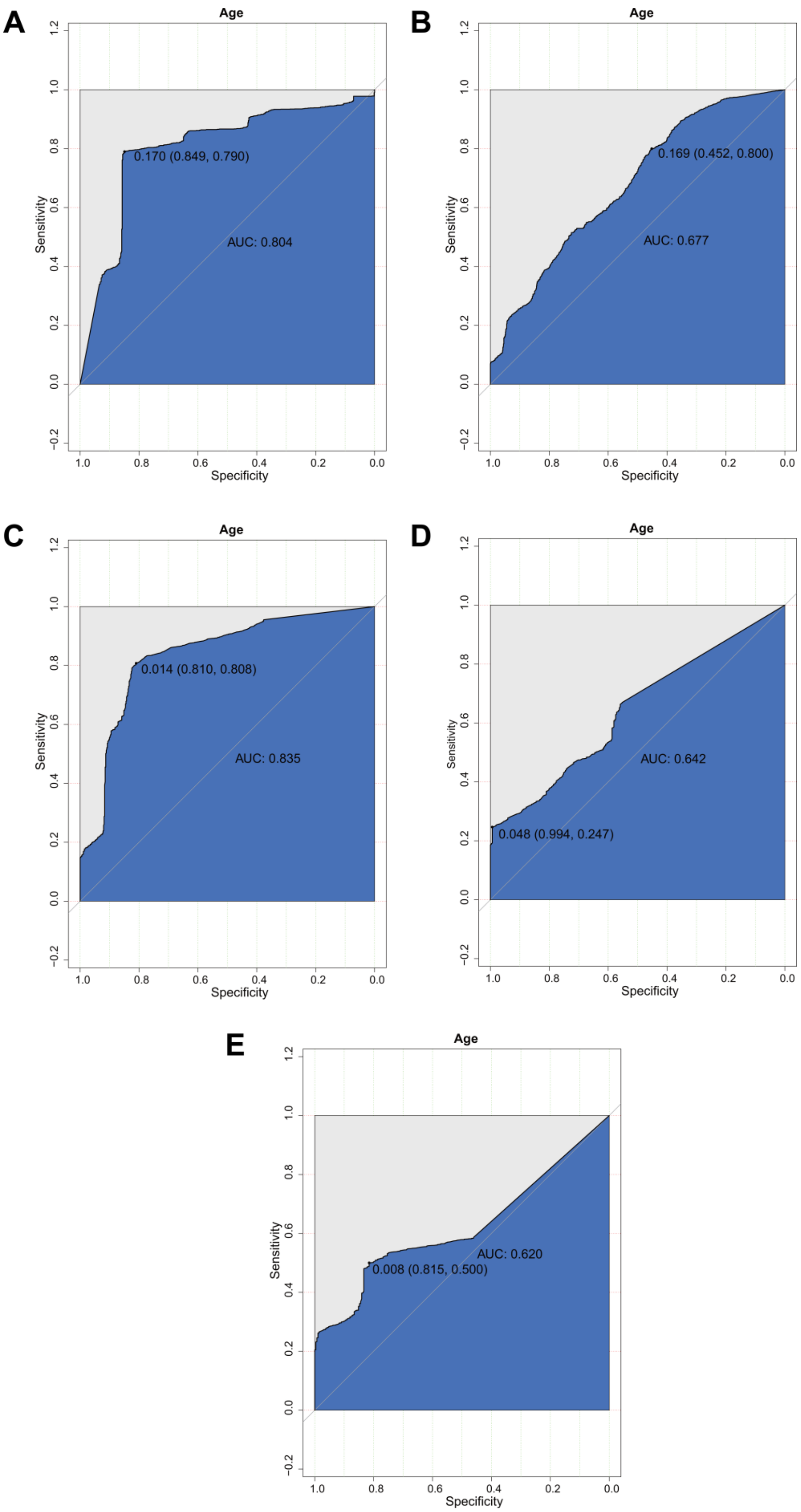


**Supplementary Figure 18. Accuracy rate of using SAL bacteria for distinguishment.**

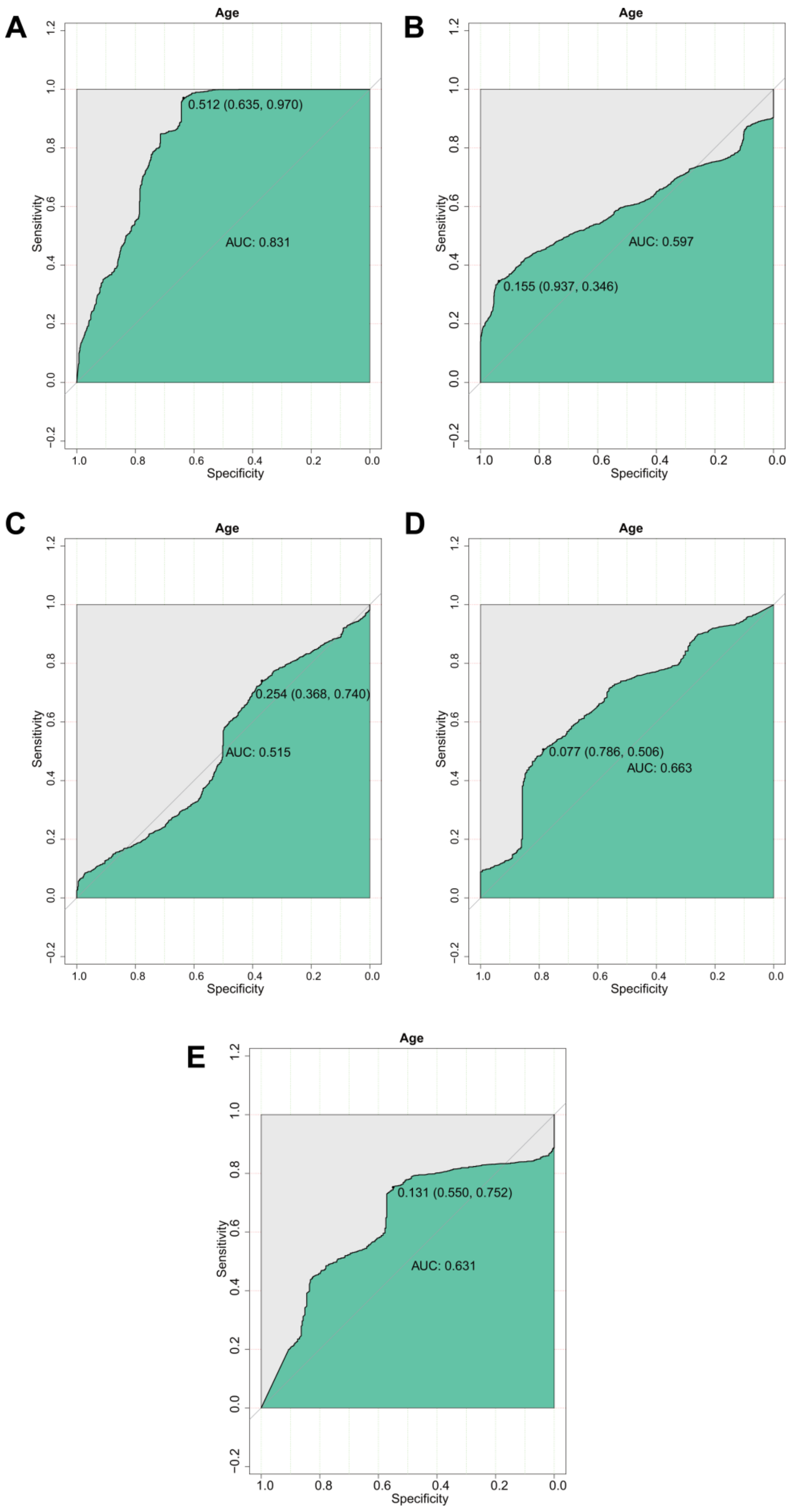


**Supplementary Figure 19. Accuracy rate of using TB bacteria for distinguishment.**
