# Supplementary material for: Microbiome succession with increasing age in three oral sites
Source: Aging (Albany NY). 2020 May 7;12(9):7874–907. doi: 10.18632/aging.103108 (PMC7244077; doi:10.18632/aging.103108)
Supplement: Supplementary Tables 1, 4, 5 [file aging-12-103108-s004..pdf]

SUPPLEMENTARY TABLES

Supplementary Table 1. All samples were collected from 3 oral sites: GCF, SAL and TB.

GCF

Groups	A_GCF	B_GCF	C_GCF	D_GCF	E_GCF
Number of people	14	20	12	7	7
Age	11:15	18:20	28:32	38:45	>50
Part	GCF	GCF	GCF	GCF	GCF
Gender	M:2;F:12	M:10;F:10	M:3;F:9	M:0;F:7	M:0;F:7

SAL

Groups	A_SAL	B_SAL	C_SAL	D_SAL	E_SAL
Number of people	14	20	12	7	6
Age	11:15	18:20	28:32	38:45	>50
Part	SAL	SAL	SAL	SAL	SAL
Gender	M:2;F:12	M:4;F:16	M:3;F:9	M:0;F:7	M:0;F:6

TB

Groups	A_TB	B_TB	C_TB	D_TB	E_TB
Number of people	14	20	12	7	7
Age	11:15	18:20	28:32	38:45	>50
Part	TB	TB	TB	TB	TB
Gender	M:2;F:12	M:4;F:16	M:3;F:9	M:0;F:7	M:0;F:7

Supplementary Table 4. STEM obtained modules in the three oral sites.

**Module 1 of GCF**			
OTU45	Firmicutes	Solobacterium	*Solobacterium_moorei*
OTU71	Firmicutes	Eubacterium	*Eubacterium_sulci*
OTU72	Firmicutes	Stomatobaculum	*Stomatobaculum_longum*
OTU102	Firmicutes	NA	NA
OTU121	Bacteroidetes	Alloprevotella	*Alloprevotella_rava*
OTU143	Firmicutes	NA	NA
OTU161	Proteobacteria	Haemophilus	NA
OTU243	Bacteroidetes	Prevotella	NA
OTU803	Firmicutes	Streptococcus	NA
OTU1545	Bacteroidetes	Prevotella	NA
OTU2142	Fusobacteria	Leptotrichia	NA
OTU2143	Fusobacteria	Leptotrichia	NA
**Module 2 of GCF**			
OTU43	Bacteroidetes	Porphyromonas	*Porphyromonas_gingivalis*
OTU52	Firmicutes	Filifactor	NA

OTU54	Firmicutes	Abiotrophia	*Abiotrophia_defectiva*
OTU56	Bacteroidetes	Porphyromonas	*Porphyromonas_endodontalis*
OTU66	Firmicutes	Parvimonas	*Parvimonas_micra*
OTU75	Fusobacteria	Leptotrichia	*Leptotrichia_goodfellowii*
OTU86	Bacteroidetes	Prevotella	NA
OTU116	Firmicutes	NA	NA
OTU123	Firmicutes	NA	NA
OTU124	Firmicutes	Dialister	*Dialister_pneumosintes*
OTU172	Actinobacteria	NA	NA
**Module 3 of GCF**			
OTU215	Firmicutes	Lactobacillus	NA
OTU20	Proteobacteria	Aggregatibacter	NA
OTU21	Proteobacteria	NA	NA
OTU49	Proteobacteria	NA	NA
OTU88	Proteobacteria	NA	NA
OTU134	Fusobacteria	Leptotrichia	*Leptotrichia_goodfellowii*
OTU746	Proteobacteria	NA	NA
**Module of SAL**			
OTU52	Firmicutes	Filifactor	NA
OTU69	Bacteroidetes	Tannerella	*Tannerella_forsythia*
OTU79	Spirochaetes	Treponema	*Treponema_medium*
OTU120	Spirochaetes	Treponema	NA
OTU124	Firmicutes	Dialister	*Dialister_pneumosintes*
OTU128	Bacteroidetes	NA	NA
OTU136	Candidatus_Saccharibacteria	NA	NA
OTU212	Firmicutes	Eubacterium	NA
OTU226	Bacteroidetes	Prevotella	NA
OTU500	Actinobacteria	Slackia	*Slackia_exigua*
OTU546	Proteobacteria	NA	NA
OTU1693	Firmicutes	NA	NA
**Module of TB**			
OTU86	Bacteroidetes	Prevotella	NA
OTU91	Bacteroidetes	NA	NA
OTU121	Bacteroidetes	Alloprevotella	*Alloprevotella_rava*
OTU272	Bacteroidetes	NA	NA
OTU546	Proteobacteria	NA	NA

OTU590	Firmicutes	Veillonella	NA
OTU770	Proteobacteria	NA	NA
OTU1693	Firmicutes	NA	NA
OTU1767	Proteobacteria	Neisseria	NA

Supplementary Table 5. Genera and species with significant difference in content between age groups. The bold names were the differential genera or species that shared by two sites.

Genus level:

GCF	SAL	TB
Acinetobacter	Bosea	Bacillus
Anaeroglobus	**Delftia**	**Bradyrhizobium**
Aquabacterium	**Escherichia-shigella**	**Brevundimonas**
Arthrobacter	Feacalibacterium	Filifactor
Bacteroides	**Herbaspiriblum**	**Pelmonas**
Bifidobacterium	**Rothia**	**Serratia**
**Bradyrhizobium**		Tannerella
**Brevundimonas**		
Buchera		
Burkholderia		
Caulobacter		
Clostridium		
Curvibacter		
**Delftia**		
Desulfovibrio		
Erythrobacter		
**Escherichia-shigella**		
Gemmiger		
**Herbaspiriblum**		
Intestinibacter		
Lachnoanaerobaculum		
Methyloversatilis		
Osclillibacter		
Paracoccus		
**Pelmonas**		
Peptostreptococcacae_ Incertae_sedis		
Propionibacterium		
Romboutsia		
Roseburia		
Ruminococcus		
**Serratia**		
Sphingomonas		
Stenotrophomonas		
Neisseria		
Oribacterium		
**Rothia**		

Solobacterium		
Stomatobaculum		
Streptococcus		

Species level:

GCF	SAL	TB
Acinetobacter_junii	***Feacalibacterium_prausnitzii***	*Eubacterium_sulci*
Acinetobacter_schindler	*Fusobacterium_mortiferum*	***Feacalibacterium_prausnitzii***
Actinalmyces_gerencseriae		*Porphyromonas_gingivalis*
Actinalmyces_massiliensis		*Tannerella_forsythia*
Anaeroglobus_geminatus		
Bifidobacterium_longum_Subsp._infantis		
Propionibacterium_acnes		
Pseudomonas_beteli		
Sphingomonas_xenophagum		
Treponema_maltophilum		
Oribacterium_sinus		
Prevotella_nanceiensis		
Solobacterium_moorei		
Stomatobaculum_longum		
