# Supplementary material for: Microbiome succession with increasing age in three oral sites
Source: Aging (Albany NY). 2020 May 7;12(9):7874–907. doi: 10.18632/aging.103108 (PMC7244077; doi:10.18632/aging.103108)
Supplement: Supplementary Table 2 [file aging-12-103108-s001..docx]

**Supplementary Table 2. Assessment of the data quality. Clean Reads refers to the sequences used for subsequent analysis after removing chimeras; AvgLen refers to the average length of Clean Reads.**

| **Sample Name** | **Clean Reads** | **AvgLen(nt)** |
| --- | --- | --- |
| A_GCF1 | 68628 | 400 |
| A_GCF2 | 63392 | 400 |
| A_GCF3 | 70067 | 400 |
| A_GCF4 | 77190 | 400 |
| A_GCF5 | 75760 | 400 |
| A_GCF6 | 77789 | 400 |
| A_GCF7 | 69821 | 400 |
| A_GCF8 | 62353 | 400 |
| A_GCF9 | 67359 | 400 |
| A_GCF10 | 61980 | 400 |
| A_GCF11 | 53106 | 400 |
| A_GCF12 | 78844 | 400 |
| A_GCF13 | 67995 | 400 |
| A_GCF14 | 69199 | 400 |
| A_SAL1 | 62251 | 400 |
| A_SAL2 | 56459 | 400 |
| A_SAL3 | 72360 | 400 |
| A_SAL4 | 76145 | 400 |
| A_SAL5 | 65978 | 400 |
| A_SAL6 | 69121 | 400 |
| A_SAL7 | 72024 | 400 |
| A_SAL8 | 67495 | 400 |
| A_SAL9 | 72364 | 400 |
| A_SAL10 | 67873 | 400 |
| A_SAL11 | 75852 | 400 |
| A_SAL12 | 74661 | 400 |
| A_SAL13 | 67995 | 400 |
| A_SAL14 | 78069 | 400 |
| A_TB1 | 80273 | 400 |
| A_TB2 | 76692 | 400 |
| A_TB3 | 69785 | 400 |
| A_TB4 | 71284 | 400 |
| A_TB5 | 69882 | 400 |
| A_TB6 | 66378 | 400 |
| A_TB7 | 67984 | 400 |
| A_TB8 | 79813 | 400 |
| A_TB9 | 66936 | 400 |
| A_TB10 | 67171 | 400 |
| A_TB11 | 33687 | 400 |
| A_TB12 | 45376 | 400 |
| A_TB13 | 74081 | 400 |
| A_TB14 | 67329 | 400 |
| B_GCF1 | 87451 | 400 |
| B_GCF2 | 91571 | 400 |
| B_GCF3 | 111472 | 400 |
| B_GCF4 | 103036 | 400 |
| B_GCF5 | 107789 | 400 |
| B_GCF6 | 109113 | 400 |
| B_GCF7 | 104845 | 400 |
| B_GCF8 | 110268 | 400 |
| B_GCF9 | 112288 | 400 |
| B_GCF10 | 100569 | 400 |
| B_GCF11 | 112847 | 400 |
| B_GCF12 | 90481 | 400 |
| B_GCF13 | 91128 | 400 |
| B_GCF14 | 110903 | 400 |
| B_GCF15 | 104305 | 400 |
| B_GCF16 | 102681 | 400 |
| B_GCF17 | 110213 | 400 |
| B_GCF18 | 87639 | 400 |
| B_GCF19 | 119364 | 400 |
| B_GCF20 | 91433 | 400 |
| B_SAL1 | 63904 | 400 |
| B_SAL2 | 70166 | 400 |
| B_SAL3 | 94063 | 400 |
| B_SAL4 | 87318 | 400 |
| B_SAL5 | 95278 | 400 |
| B_SAL6 | 96563 | 400 |
| B_SAL7 | 102641 | 400 |
| B_SAL8 | 99743 | 400 |
| B_SAL9 | 107103 | 400 |
| B_SAL10 | 102839 | 400 |
| B_SAL11 | 99012 | 400 |
| B_SAL12 | 59751 | 400 |
| B_SAL13 | 70333 | 400 |
| B_SAL14 | 68643 | 400 |
| B_SAL15 | 66848 | 400 |
| B_SAL16 | 70778 | 400 |
| B_SAL17 | 60855 | 400 |
| B_SAL18 | 60184 | 400 |
| B_SAL19 | 67796 | 400 |
| B_SAL20 | 74243 | 400 |
| B_TB1 | 65762 | 400 |
| B_TB2 | 69175 | 400 |
| B_TB3 | 70679 | 400 |
| B_TB4 | 64153 | 400 |
| B_TB5 | 70009 | 400 |
| B_TB6 | 58858 | 400 |
| B_TB7 | 104869 | 400 |
| B_TB8 | 69422 | 400 |
| B_TB9 | 72865 | 400 |
| B_TB10 | 71457 | 400 |
| B_TB11 | 77365 | 400 |
| B_TB12 | 67282 | 400 |
| B_TB13 | 70737 | 400 |
| B_TB14 | 63406 | 400 |
| B_TB15 | 61402 | 400 |
| B_TB16 | 57990 | 400 |
| B_TB17 | 56950 | 400 |
| B_TB18 | 60529 | 400 |
| B_TB19 | 61309 | 400 |
| B_TB20 | 63410 | 400 |
| C_GCF1 | 112794 | 400 |
| C_GCF2 | 108319 | 400 |
| C_GCF3 | 110843 | 400 |
| C_GCF4 | 112217 | 400 |
| C_GCF5 | 132490 | 400 |
| C_GCF6 | 108794 | 400 |
| C_GCF7 | 138091 | 400 |
| C_GCF8 | 114000 | 400 |
| C_GCF9 | 81189 | 400 |
| C_GCF10 | 118657 | 400 |
| C_GCF11 | 100293 | 400 |
| C_GCF12 | 107575 | 400 |
| C_SAL1 | 69171 | 400 |
| C_SAL2 | 110015 | 400 |
| C_SAL3 | 104721 | 400 |
| C_SAL4 | 103588 | 400 |
| C_SAL5 | 65279 | 400 |
| C_SAL6 | 70112 | 400 |
| C_SAL7 | 73926 | 400 |
| C_SAL8 | 76859 | 400 |
| C_SAL9 | 72028 | 400 |
| C_SAL10 | 68378 | 400 |
| C_SAL11 | 104652 | 400 |
| C_SAL12 | 106728 | 400 |
| C_TB1 | 60602 | 400 |
| C_TB2 | 104788 | 400 |
| C_TB3 | 106505 | 400 |
| C_TB4 | 101041 | 400 |
| C_TB5 | 57908 | 400 |
| C_TB6 | 64403 | 400 |
| C_TB7 | 70687 | 400 |
| C_TB8 | 71531 | 400 |
| C_TB9 | 63220 | 400 |
| C_TB10 | 57433 | 400 |
| C_TB11 | 99641 | 400 |
| C_TB12 | 64736 | 400 |
| D_GCF1 | 111702 | 400 |
| D_GCF2 | 114118 | 400 |
| D_GCF3 | 113789 | 400 |
| D_GCF4 | 118781 | 400 |
| D_GCF5 | 96637 | 400 |
| D_GCF6 | 105905 | 400 |
| D_GCF7 | 103091 | 400 |
| D_SAL1 | 79657 | 400 |
| D_SAL2 | 60564 | 400 |
| D_SAL3 | 76624 | 400 |
| D_SAL4 | 65523 | 400 |
| D_SAL5 | 111461 | 400 |
| D_SAL6 | 100687 | 400 |
| D_SAL7 | 105057 | 400 |
| D_TB1 | 66520 | 400 |
| D_TB2 | 64007 | 400 |
| D_TB3 | 63652 | 400 |
| D_TB4 | 62521 | 400 |
| D_TB5 | 111413 | 400 |
| D_TB6 | 69593 | 400 |
| D_TB7 | 106693 | 400 |
| E_GCF1 | 87914 | 400 |
| E_GCF2 | 108860 | 400 |
| E_GCF3 | 100608 | 400 |
| E_GCF4 | 107089 | 400 |
| E_GCF5 | 104304 | 400 |
| E_GCF6 | 109882 | 400 |
| E_GCF7 | 108100 | 400 |
| E_SAL1 | 112785 | 400 |
| E_SAL2 | 112246 | 400 |
| E_SAL3 | 105099 | 400 |
| E_SAL4 | 103855 | 400 |
| E_SAL5 | 97729 | 400 |
| E_SAL6 | 102411 | 400 |
| E_TB1 | 110533 | 400 |
| E_TB2 | 78404 | 400 |
| E_TB3 | 78093 | 400 |
| E_TB4 | 68085 | 400 |
| E_TB5 | 72647 | 400 |
| E_TB6 | 68708 | 400 |
| E_TB7 | 99660 | 400 |
