# Supplementary material for: Microbiome succession with increasing age in three oral sites
Source: Aging (Albany NY). 2020 May 7;12(9):7874–907. doi: 10.18632/aging.103108 (PMC7244077; doi:10.18632/aging.103108)
Supplement: Supplementary Table 3 [file aging-12-103108-s002..docx]

**Supplementary Table 3. Annotated microbiota to different levels of bacterial taxonomic units.**

| **Sample Name** | **OTUs** | **Species** | **Genus** | **Family** | **Order** | **Class** | **Phylum** |
| --- | --- | --- | --- | --- | --- | --- | --- |
| A_GCF01 | 728 | 144 | 211 | 133 | 69 | 38 | 18 |
| A_GCF02 | 316 | 87 | 112 | 71 | 38 | 23 | 11 |
| A_GCF03 | 503 | 125 | 170 | 106 | 53 | 30 | 16 |
| A_GCF04 | 485 | 120 | 155 | 91 | 48 | 30 | 16 |
| A_GCF05 | 485 | 128 | 157 | 102 | 58 | 31 | 17 |
| A_GCF06 | 305 | 83 | 105 | 77 | 40 | 25 | 13 |
| A_GCF07 | 364 | 113 | 118 | 67 | 34 | 20 | 11 |
| A_GCF08 | 401 | 119 | 134 | 79 | 40 | 23 | 12 |
| A_GCF09 | 191 | 64 | 66 | 47 | 28 | 18 | 11 |
| A_GCF10 | 812 | 178 | 209 | 119 | 64 | 35 | 17 |
| A_GCF11 | 1027 | 201 | 249 | 138 | 67 | 38 | 19 |
| A_GCF12 | 499 | 146 | 162 | 90 | 43 | 25 | 13 |
| A_GCF13 | 402 | 123 | 129 | 76 | 39 | 23 | 12 |
| A_GCF14 | 464 | 130 | 144 | 88 | 49 | 26 | 13 |
| A_SAL01 | 545 | 125 | 166 | 109 | 53 | 32 | 16 |
| A_SAL02 | 350 | 112 | 123 | 79 | 42 | 25 | 12 |
| A_SAL03 | 505 | 117 | 155 | 110 | 59 | 31 | 14 |
| A_SAL04 | 411 | 115 | 128 | 82 | 43 | 25 | 14 |
| A_SAL05 | 329 | 109 | 99 | 66 | 34 | 18 | 10 |
| A_SAL06 | 384 | 100 | 131 | 86 | 41 | 25 | 13 |
| A_SAL07 | 313 | 104 | 105 | 57 | 30 | 19 | 11 |
| A_SAL08 | 257 | 84 | 85 | 57 | 30 | 17 | 10 |
| A_SAL09 | 262 | 85 | 93 | 61 | 31 | 18 | 10 |
| A_SAL10 | 827 | 175 | 220 | 129 | 64 | 33 | 15 |
| A_SAL11 | 637 | 177 | 197 | 104 | 56 | 30 | 17 |
| A_SAL12 | 444 | 133 | 142 | 83 | 45 | 27 | 16 |
| A_SAL13 | 402 | 123 | 129 | 76 | 39 | 23 | 12 |
| A_SAL14 | 528 | 155 | 160 | 89 | 44 | 25 | 16 |
| A_TB01 | 416 | 107 | 133 | 86 | 45 | 27 | 15 |
| A_TB02 | 521 | 136 | 142 | 86 | 48 | 26 | 16 |
| A_TB03 | 593 | 135 | 177 | 109 | 58 | 32 | 14 |
| A_TB04 | 525 | 112 | 167 | 110 | 59 | 32 | 15 |
| A_TB05 | 568 | 137 | 170 | 96 | 55 | 34 | 17 |
| A_TB06 | 257 | 83 | 98 | 59 | 32 | 17 | 9 |
| A_TB07 | 435 | 99 | 138 | 89 | 45 | 27 | 12 |
| A_TB08 | 237 | 81 | 76 | 55 | 27 | 19 | 11 |
| A_TB09 | 254 | 84 | 87 | 58 | 31 | 19 | 11 |
| A_TB10 | 233 | 82 | 85 | 57 | 31 | 17 | 9 |
| A_TB11 | 772 | 165 | 211 | 120 | 63 | 35 | 17 |
| A_TB12 | 780 | 192 | 229 | 118 | 61 | 32 | 15 |
| A_TB13 | 262 | 89 | 93 | 59 | 34 | 17 | 9 |
| A_TB14 | 357 | 114 | 115 | 69 | 36 | 21 | 12 |
| B_GCF01 | 297 | 94 | 81 | 55 | 32 | 19 | 11 |
| B_GCF02 | 546 | 150 | 157 | 93 | 55 | 28 | 14 |
| B_GCF03 | 594 | 147 | 144 | 91 | 48 | 26 | 15 |
| B_GCF04 | 452 | 121 | 124 | 81 | 45 | 24 | 14 |
| B_GCF05 | 571 | 149 | 149 | 98 | 52 | 29 | 17 |
| B_GCF06 | 835 | 170 | 197 | 110 | 62 | 33 | 17 |
| B_GCF07 | 413 | 123 | 138 | 83 | 46 | 26 | 16 |
| B_GCF08 | 610 | 150 | 179 | 115 | 61 | 32 | 15 |
| B_GCF09 | 621 | 155 | 187 | 115 | 62 | 33 | 15 |
| B_GCF10 | 638 | 165 | 194 | 115 | 60 | 30 | 15 |
| B_GCF11 | 567 | 144 | 159 | 102 | 54 | 29 | 16 |
| B_GCF12 | 386 | 115 | 108 | 61 | 37 | 22 | 12 |
| B_GCF13 | 252 | 81 | 74 | 50 | 30 | 21 | 13 |
| B_GCF14 | 603 | 140 | 160 | 91 | 55 | 29 | 15 |
| B_GCF15 | 421 | 113 | 119 | 73 | 37 | 24 | 14 |
| B_GCF16 | 417 | 124 | 117 | 76 | 41 | 21 | 13 |
| B_GCF17 | 414 | 122 | 128 | 75 | 42 | 24 | 15 |
| B_GCF18 | 446 | 126 | 123 | 79 | 43 | 26 | 13 |
| B_GCF19 | 393 | 118 | 121 | 73 | 40 | 25 | 15 |
| B_GCF20 | 490 | 132 | 134 | 78 | 49 | 28 | 14 |
| B_SAL01 | 435 | 99 | 120 | 86 | 47 | 28 | 13 |
| B_SAL10 | 291 | 91 | 71 | 51 | 31 | 21 | 13 |
| B_SAL11 | 324 | 95 | 82 | 58 | 32 | 20 | 12 |
| B_SAL12 | 518 | 145 | 143 | 78 | 39 | 23 | 12 |
| B_SAL13 | 247 | 84 | 72 | 48 | 25 | 18 | 10 |
| B_SAL14 | 478 | 134 | 136 | 72 | 38 | 23 | 13 |
| B_SAL15 | 544 | 143 | 133 | 68 | 34 | 20 | 12 |
| B_SAL16 | 461 | 134 | 128 | 72 | 35 | 22 | 13 |
| B_SAL17 | 469 | 128 | 130 | 76 | 38 | 23 | 13 |
| B_SAL18 | 351 | 105 | 103 | 59 | 32 | 19 | 11 |
| B_SAL19 | 329 | 89 | 94 | 61 | 34 | 21 | 12 |
| B_SAL2 | 363 | 99 | 101 | 64 | 32 | 20 | 12 |
| B_SAL20 | 370 | 96 | 99 | 62 | 32 | 19 | 11 |
| B_SAL3 | 350 | 99 | 98 | 69 | 35 | 21 | 12 |
| B_SAL4 | 302 | 89 | 86 | 59 | 35 | 21 | 12 |
| B_SAL5 | 353 | 107 | 105 | 64 | 38 | 21 | 12 |
| B_SAL6 | 348 | 102 | 101 | 67 | 36 | 21 | 11 |
| B_SAL7 | 375 | 102 | 104 | 73 | 40 | 23 | 14 |
| B_SAL8 | 610 | 144 | 166 | 110 | 57 | 30 | 15 |
| B_SAL9 | 292 | 93 | 72 | 49 | 27 | 19 | 11 |
| B_TB02 | 258 | 78 | 76 | 55 | 31 | 21 | 13 |
| B_TB03 | 201 | 67 | 62 | 43 | 28 | 19 | 11 |
| B_TB04 | 232 | 74 | 66 | 49 | 29 | 19 | 11 |
| B_TB05 | 265 | 88 | 83 | 54 | 31 | 20 | 12 |
| B_TB06 | 211 | 62 | 59 | 45 | 27 | 20 | 12 |
| B_TB07 | 452 | 136 | 136 | 82 | 41 | 23 | 13 |
| B_TB08 | 158 | 52 | 49 | 36 | 22 | 17 | 9 |
| B_TB09 | 200 | 62 | 55 | 42 | 26 | 18 | 10 |
| B_TB1 | 185 | 60 | 54 | 38 | 22 | 17 | 9 |
| B_TB10 | 228 | 73 | 57 | 40 | 24 | 19 | 11 |
| B_TB11 | 208 | 74 | 55 | 42 | 25 | 18 | 10 |
| B_TB12 | 191 | 66 | 58 | 42 | 25 | 18 | 10 |
| B_TB13 | 196 | 70 | 57 | 41 | 25 | 19 | 11 |
| B_TB14 | 175 | 63 | 58 | 41 | 26 | 19 | 11 |
| B_TB15 | 292 | 104 | 97 | 57 | 29 | 20 | 12 |
| B_TB16 | 170 | 53 | 54 | 40 | 23 | 17 | 10 |
| B_TB17 | 183 | 63 | 57 | 43 | 27 | 19 | 11 |
| B_TB18 | 200 | 66 | 71 | 48 | 29 | 18 | 10 |
| B_TB19 | 269 | 88 | 80 | 55 | 32 | 21 | 13 |
| B_TB20 | 330 | 103 | 96 | 60 | 37 | 21 | 13 |
| C_GCF01 | 876 | 145 | 183 | 120 | 70 | 42 | 20 |
| C_GCF02 | 430 | 127 | 142 | 83 | 43 | 23 | 14 |
| C_GCF03 | 207 | 71 | 64 | 46 | 28 | 20 | 12 |
| C_GCF04 | 235 | 80 | 68 | 49 | 27 | 18 | 10 |
| C_GCF05 | 415 | 107 | 120 | 79 | 49 | 29 | 15 |
| C_GCF06 | 378 | 110 | 110 | 67 | 37 | 22 | 13 |
| C_GCF07 | 584 | 142 | 154 | 93 | 58 | 31 | 17 |
| C_GCF08 | 568 | 145 | 151 | 97 | 53 | 27 | 15 |
| C_GCF09 | 421 | 113 | 117 | 73 | 36 | 21 | 12 |
| C_GCF10 | 404 | 112 | 119 | 76 | 41 | 26 | 16 |
| C_GCF11 | 289 | 95 | 88 | 62 | 33 | 20 | 12 |
| C_GCF12 | 314 | 97 | 89 | 65 | 34 | 20 | 12 |
| C_SAL01 | 393 | 86 | 99 | 77 | 43 | 27 | 15 |
| C_SAL02 | 315 | 98 | 82 | 53 | 29 | 20 | 12 |
| C_SAL03 | 319 | 96 | 90 | 61 | 30 | 21 | 13 |
| C_SAL04 | 272 | 86 | 72 | 47 | 28 | 19 | 11 |
| C_SAL05 | 432 | 95 | 119 | 80 | 45 | 27 | 16 |
| C_SAL06 | 424 | 105 | 116 | 82 | 45 | 27 | 15 |
| C_SAL07 | 378 | 102 | 109 | 75 | 48 | 26 | 14 |
| C_SAL08 | 449 | 107 | 122 | 85 | 47 | 28 | 15 |
| C_SAL09 | 463 | 102 | 119 | 90 | 51 | 27 | 14 |
| C_SAL10 | 477 | 102 | 117 | 88 | 44 | 27 | 13 |
| C_SAL11 | 292 | 84 | 72 | 50 | 26 | 20 | 12 |
| C_SAL12 | 399 | 116 | 135 | 83 | 42 | 23 | 14 |
| C_TB01 | 202 | 63 | 66 | 45 | 27 | 19 | 11 |
| C_TB02 | 294 | 91 | 87 | 59 | 31 | 19 | 11 |
| C_TB03 | 229 | 79 | 72 | 49 | 27 | 18 | 10 |
| C_TB04 | 356 | 109 | 124 | 80 | 41 | 27 | 14 |
| C_TB05 | 179 | 55 | 55 | 39 | 24 | 18 | 10 |
| C_TB06 | 181 | 60 | 57 | 41 | 24 | 16 | 9 |
| C_TB07 | 294 | 98 | 90 | 52 | 27 | 19 | 11 |
| C_TB08 | 202 | 66 | 63 | 46 | 27 | 18 | 10 |
| C_TB09 | 301 | 107 | 95 | 54 | 28 | 19 | 11 |
| C_TB10 | 199 | 64 | 57 | 40 | 24 | 18 | 10 |
| C_TB11 | 274 | 89 | 78 | 53 | 30 | 21 | 13 |
| C_TB12 | 252 | 79 | 63 | 46 | 27 | 21 | 13 |
| D_GCF01 | 486 | 113 | 118 | 84 | 49 | 27 | 14 |
| D_GCF02 | 458 | 109 | 123 | 85 | 50 | 29 | 14 |
| D_GCF03 | 451 | 124 | 123 | 76 | 44 | 25 | 14 |
| D_GCF04 | 405 | 101 | 105 | 69 | 39 | 22 | 13 |
| D_GCF05 | 376 | 115 | 115 | 76 | 43 | 25 | 15 |
| D_GCF06 | 196 | 68 | 63 | 50 | 27 | 20 | 12 |
| D_GCF07 | 214 | 66 | 62 | 47 | 25 | 18 | 10 |
| D_SAL01 | 419 | 98 | 115 | 88 | 47 | 30 | 16 |
| D_SAL02 | 341 | 92 | 88 | 67 | 39 | 24 | 12 |
| D_SAL03 | 398 | 101 | 116 | 85 | 48 | 26 | 15 |
| D_SAL04 | 433 | 93 | 117 | 88 | 53 | 31 | 16 |
| D_SAL05 | 311 | 91 | 80 | 50 | 30 | 20 | 12 |
| D_SAL06 | 372 | 110 | 111 | 72 | 39 | 24 | 14 |
| D_SAL07 | 419 | 119 | 116 | 75 | 41 | 23 | 13 |
| D_TB01 | 282 | 97 | 84 | 47 | 25 | 18 | 10 |
| D_TB02 | 184 | 56 | 58 | 44 | 25 | 19 | 11 |
| D_TB03 | 180 | 54 | 52 | 39 | 24 | 18 | 11 |
| D_TB04 | 274 | 89 | 90 | 52 | 29 | 20 | 11 |
| D_TB05 | 289 | 91 | 78 | 56 | 33 | 22 | 13 |
| D_TB06 | 265 | 87 | 62 | 47 | 28 | 20 | 12 |
| D_TB07 | 298 | 91 | 85 | 57 | 30 | 21 | 13 |
| E_GCF01 | 219 | 67 | 62 | 43 | 25 | 15 | 8 |
| E_GCF02 | 214 | 70 | 65 | 46 | 29 | 19 | 11 |
| E_GCF03 | 249 | 86 | 69 | 51 | 31 | 20 | 12 |
| E_GCF04 | 236 | 80 | 67 | 45 | 27 | 20 | 12 |
| E_GCF05 | 228 | 80 | 73 | 52 | 31 | 19 | 11 |
| E_GCF06 | 216 | 74 | 67 | 46 | 26 | 18 | 10 |
| E_GCF07 | 210 | 65 | 60 | 43 | 25 | 18 | 10 |
| E_SAL01 | 343 | 110 | 90 | 61 | 31 | 19 | 11 |
| E_SAL02 | 361 | 103 | 95 | 61 | 33 | 20 | 12 |
| E_SAL03 | 428 | 124 | 118 | 77 | 39 | 24 | 14 |
| E_SAL04 | 237 | 77 | 63 | 47 | 27 | 19 | 11 |
| E_SAL05 | 301 | 95 | 80 | 56 | 30 | 19 | 11 |
| E_SAL06 | 257 | 86 | 72 | 53 | 29 | 20 | 12 |
| E_TB01 | 296 | 93 | 85 | 61 | 33 | 22 | 14 |
| E_TB02 | 244 | 78 | 65 | 47 | 29 | 21 | 13 |
| E_TB03 | 182 | 62 | 55 | 40 | 25 | 19 | 11 |
| E_TB04 | 192 | 60 | 51 | 39 | 25 | 18 | 10 |
| E_TB05 | 220 | 69 | 54 | 39 | 25 | 20 | 12 |
| E_TB06 | 220 | 68 | 54 | 41 | 24 | 18 | 10 |
| E_TB07 | 278 | 94 | 79 | 56 | 33 | 20 | 12 |
