# Supplementary material for: Microbiome succession with increasing age in three oral sites
Source: Aging (Albany NY). 2020 May 7;12(9):7874–907. doi: 10.18632/aging.103108 (PMC7244077; doi:10.18632/aging.103108)
Supplement: Supplementary Table 6 [file aging-12-103108-s003..docx]

**Supplementary Table 6. Genera and species with significant difference in content between oral sites in an age.**

**Genus level:**

| A | B | C | D | E |
| --- | --- | --- | --- | --- |
| Corynebacterium | Abiotrophia | Abiotrophia | Actinomyces | Abiotrophia |
| Eubacterium | Acinetobacter | Aggregatibacter | Bacillus | Actinomyces |
| Lachnoanaerobaculum | Aggregatibacter | Alloprevotella | Bradyrhizobium | Aggregatibacter |
| Neisseria | Aminobacterium | Atopobium | Butyricimonas | Capnocytophaga |
| Oribacterium | Anaeroglobus | Bradyrhizobium | Capnocytophaga | Gemella |
| Parvimonas | Anaerostipes | Butyricimonas | Gemella | Granulicatella |
| Peptostreptococcus | Atopobium | Capnocytophaga | Granulicatella | Lautropia |
| Prevotella | Bradyrhizobium | Corynebacterium | Lachnoanaerobaculum | Mycoplasma |
| Rothia | Capnocytophaga | Eikenella | Lactobacillus | **Solobacterium** |
| Solobacterium | Cardiobacterium | Eubacterium | Megasphaera |  |
| Sphingobium | Catonella | Gemella | Mycoplasma |  |
| Streptococcus | Clostridium_sensu_stricto | Gp1 | Oribacterium |  |
|  | Clostridium_XlVa | Gp2 | **Solobacterium** |  |
|  | Corynebacterium | Granulicatella | Stomatobaculum |  |
|  | Deinococcus | Megasphaera | Streptobacillus |  |
|  | Delftia | Oribacterium | Streptococcus |  |
|  | Desulfomicrobium | Parvimonas | Streptomyces |  |
|  | Dialister | Prevotella | Treponema |  |
|  | Eikenella | Ralstonia |  |  |
|  | Faecalibacterium | Selenomonas |  |  |
|  | Filifactor | **Solobacterium** |  |  |
|  | Fretibacterium | Sphingomonas |  |  |
|  | Fusicatenibacter | Stomatobaculum |  |  |
|  | Gemella | Streptobacillus |  |  |
|  | Granulicatella | Streptococcus |  |  |
|  | Intestinibacter | Streptomyces |  |  |
|  | Lachnoanaerobaculum | Treponema |  |  |
|  | Lactobacillus | Veillonella |  |  |
|  | Lautropia |  |  |  |
|  | Massilia |  |  |  |
|  | Megasphaera |  |  |  |
|  | Micrococcus |  |  |  |
|  | Mycoplasma |  |  |  |
|  | Neisseria |  |  |  |
|  | Novosphingobium |  |  |  |
|  | Oribacterium |  |  |  |
|  | Peptostreptococcaceae_  incertae_sedis |  |  |  |
|  | Peptostreptococcus |  |  |  |
|  | Prevotella |  |  |  |
|  | Ralstonia |  |  |  |
|  | Romboutsia |  |  |  |
|  | Rothia |  |  |  |
|  | Saccharibacteria_genera_  incertae_sedis |  |  |  |
|  | Selenomonas |  |  |  |
|  | **Solobacterium** |  |  |  |
|  | Sphingomonas |  |  |  |
|  | Sporanaerobacter |  |  |  |
|  | Stomatobaculum |  |  |  |
|  | Streptococcus |  |  |  |
|  | Streptophyta |  |  |  |
|  | Syntrophomonas |  |  |  |
|  | Tannerella |  |  |  |
|  | Treponema |  |  |  |
|  | Turicibacter |  |  |  |
|  | Veillonella |  |  |  |
|  | Wolinella |  |  |  |

**Species level**

| **A** | **B** | **C** | **D** | **E** |
| --- | --- | --- | --- | --- |
| *Actinomyces_graevenitzii* | *Abiotrophia_defectiva* | *Abiotrophia_defectiva* | ***Actinomyces_odontolyticus*** | *Abiotrophia_defectiva* |
| *Actinomyces_massiliensis* | *Acinetobacter_baumannii* | *Actinomyces_graevenitzii* | *Bacillus_thermoamylovorans* | *Actinomyces_graevenitzii* |
| ***Actinomyces_odontolyticus*** | *Acinetobacter_junii* | ***Actinomyces_odontolyticus*** | *Bacteroides_uniformis* | ***Actinomyces_odontolyticus*** |
| *Eubacterium_sulci* | *Acinetobacter_schindleri* | *Alloprevotella_rava* | *Butyricimonas_virosa* | *Anaeroglobus_geminatus* |
| *Oribacterium_sinus* | *Actinomyces_gerencseriae* | *Alloprevotella_tannerae* | *Campylobacter_gracilis* | *Bacteroides_coprocola* |
| *Parvimonas_micra* | *Actinomyces_graevenitzii* | *Butyricimonas_virosa* | *Eubacterium_sulci* | *Campylobacter_gracilis* |
| *Peptostreptococcus_stomatis* | *Actinomyces_massiliensis* | *Campylobacter_gracilis* | *Gemella_haemolysans* | *Capnocytophaga_leadbetteri* |
| *Prevotella_jejuni* | ***Actinomyces_odontolyticus*** | *Capnocytophaga_granulosa* | *Granulicatella_elegans* | *Capnocytophaga_ochracea* |
| *Prevotella_pallens* | *Alloprevotella_rava* | *Capnocytophaga_leadbetteri* | *Mycoplasma_faucium* | *Capnocytophaga_sputigena* |
| ***Solobacterium_moorei*** | *Aminobacterium_thunnarium* | *Capnocytophaga_ochracea* | *Oribacterium_sinus* | *Eubacterium_sulci* |
|  | *Anaeroglobus_geminatus* | *Capnocytophaga_sputigena* | *Porphyromonas_catoniae* | *Gemella_haemolysans* |
|  | *Campylobacter_gracilis* | *Cardiobacterium_valvarum* | *Prevotella_nanceiensis* | *Granulicatella_elegans* |
|  | *Capnocytophaga_granulosa* | *Corynebacterium_durum* | ***Solobacterium_moorei*** | *Lautropia_mirabilis* |
|  | *Capnocytophaga_leadbetteri* | *Dialister_invisus* | *Stomatobaculum_longum* | *Leptotrichia_shahii* |
|  | *Capnocytophaga_ochracea* | *Eikenella_corrodens* | *Treponema_amylovorum* | *Mycoplasma_faucium* |
|  | *Capnocytophaga_sputigena* | *Eubacterium_sulci* | *Treponema_medium* | *Neisseria_oralis* |
|  | *Cardiobacterium_hominis* | *Gemella_haemolysans* | *Veillonella_dispar* | *Prevotella_micans* |
|  | *Cardiobacterium_valvarum* | *Granulicatella_elegans* |  | *Ralstonia_pickettii* |
|  | *Catonella_morbi* | *Leptotrichia_goodfellowii* |  | *Ruminococcus_bromii* |
|  | *Corynebacterium_durum* | *Leptotrichia_hongkongensis* |  | ***Solobacterium_moorei*** |
|  | *Desulfomicrobium_orale* | *Megasphaera_micronuciformis* |  | *Streptococcus_intermedius* |
|  | *Dialister_invisus* | *Neisseria_oralis* |  | *Streptococcus_mutans* |
|  | *Dialister_pneumosintes* | *Oribacterium_sinus* |  | *Tannerella_forsythia* |
|  | *Eikenella_corrodens* | *Parvimonas_micra* |  | *Treponema_amylovorum* |
|  | *Faecalibacterium_prausnitzii* | *Prevotella_aurantiaca* |  | *Treponema_medium* |
|  | *Fretibacterium_fastidiosum* | *Prevotella_copri* |  |  |
|  | *Fusicatenibacter_saccharivorans* | *Prevotella_jejuni* |  |  |
|  | *Gemella_haemolysans* | *Prevotella_loescheii* |  |  |
|  | *Granulicatella_elegans* | *Prevotella_maculosa* |  |  |
|  | *Haemophilus_parainfluenzae* | *Prevotella_nanceiensis* |  |  |
|  | *Intestinibacter_bartlettii* | *Prevotella_pleuritidis* |  |  |
|  | *Lautropia_mirabilis* | *Prevotella_saccharolytica* |  |  |
|  | *Leptotrichia_hongkongensis* | *Prevotella_salivae* |  |  |
|  | *Leptotrichia_shahii* | *Ralstonia_pickettii* |  |  |
|  | *Megasphaera_micronuciformis* | *Selenomonas_artemidis* |  |  |
|  | *Mycoplasma_faucium* | *Selenomonas_infelix* |  |  |
|  | *Neisseria_oralis* | *Selenomonas_noxia* |  |  |
|  | *Oribacterium_sinus* | ***Solobacterium_moorei*** |  |  |
|  | *Peptostreptococcus_stomatis* | *Stomatobaculum_longum* |  |  |
|  | *Porphyromonas_endodontalis* | *Treponema_amylovorum* |  |  |
|  | *Porphyromonas_gingivalis* | *Treponema_denticola* |  |  |
|  | *Prevotella_aurantiaca* | *Treponema_medium* |  |  |
|  | *Prevotella_baroniae* | *Veillonella_dispar* |  |  |
|  | *Prevotella_denticola* |  |  |  |
|  | *Prevotella_jejuni* |  |  |  |
|  | *Prevotella_loescheii* |  |  |  |
|  | *Prevotella_maculosa* |  |  |  |
|  | *Prevotella_micans* |  |  |  |
|  | *Prevotella_nanceiensis* |  |  |  |
|  | *Prevotella_nigrescens* |  |  |  |
|  | *Prevotella_pallens* |  |  |  |
|  | *Prevotella_pleuritidis* |  |  |  |
|  | *Prevotella_saccharolytica* |  |  |  |
|  | *Prevotella_salivae* |  |  |  |
|  | *Prevotella_shahii* |  |  |  |
|  | *Ralstonia_pickettii* |  |  |  |
|  | *Selenomonas_artemidis* |  |  |  |
|  | *Selenomonas_infelix* |  |  |  |
|  | *Selenomonas_noxia* |  |  |  |
|  | *Selenomonas_sputigena* |  |  |  |
|  | ***Solobacterium_moorei*** |  |  |  |
|  | *Sporanaerobacter_acetigenes* |  |  |  |
|  | *Stomatobaculum_longum* |  |  |  |
|  | *Streptococcus_intermedius* |  |  |  |
|  | *Tannerella_forsythia* |  |  |  |
|  | *Treponema_amylovorum* |  |  |  |
|  | *Treponema_denticola* |  |  |  |
|  | *Treponema_lecithinolyticum* |  |  |  |
|  | *Treponema_medium* |  |  |  |
|  | *Turicibacter_sanguinis* |  |  |  |
|  | *Veillonella_dispar* |  |  |  |
|  | *Wolinella_succinogenes* |  |  |  |
